# Equity, community, and accountability: Leveraging a department-level climate survey as a tool for action

**DOI:** 10.1371/journal.pone.0290065

**Published:** 2023-08-17

**Authors:** Gabriel M. Barrile, Riley F. Bernard, Rebecca C. Wilcox, Justine A. Becker, Michael E. Dillon, Rebecca R. Thomas-Kuzilik, Sara P. Bombaci, Bethann Garramon Merkle

**Affiliations:** 1 Department of Zoology and Physiology, University of Wyoming, Laramie, Wyoming, United States of America; 2 Department of Ecology, Montana State University, Bozeman, Montana, United States of America; 3 Department of Fish, Wildlife, and Conservation Biology, Colorado State University, Fort Collins, Colorado, United States of America; Lamar University, UNITED STATES

## Abstract

Organizational climate is a key determinant of diverse aspects of success in work settings, including in academia. Power dynamics in higher education can result in inequitable experiences of workplace climate, potentially harming the well-being and productivity of employees. Quantifying experiences of climate across employment categories can help identify changes necessary to create a more equitable workplace for all. We developed and administered a climate survey within our academic workplace—the Department of Zoology and Physiology at the University of Wyoming—to evaluate experiences of climate across three employment categories: faculty, graduate students, and staff. Our survey included a combination of closed-response (e.g., Likert-scale) and open-ended questions. Most department members (82%) completed the survey, which was administered in fall 2021. Faculty generally reported more positive experiences than staff. Graduate students often fell between these two groups, though in some survey sections (e.g., mental health and well-being) students reported the most negative experiences of departmental climate. Three common themes emerged from the analysis of open-ended responses: equity, community, and accountability. We discuss how these themes correspond to concrete action items for improving our departmental climate, some of which have been implemented already, while others constitute future initiatives and/or require a collective push towards systemic change in academia. Finally, service work of this type often falls outside of job descriptions, requiring individuals to either work more or trade-off productivity in other areas that are formally evaluated. With the goal of minimizing this burden for others, we detail our process and provide the materials and framework necessary to streamline this process for other departments aiming to evaluate workplace climate as a key first step in building a positive work environment for all employees.

## Introduction

The overall experience of employees in the workplace often is referred to as organizational climate (hereafter, climate) [1–3]. Climate is a key determinant of diverse aspects of success in work settings [4–6]. In higher education, for instance, climate positively correlates with measures of performance and productivity, such as the number of publications and grants received [7–9]. Yet, individuals from marginalized groups (e.g., gender and racial minorities) and those in positions with less privilege (e.g., junior faculty, graduate students, staff) often face discrimination and structural biases within their institutions, leading to inequitable and negative experiences within the workplace [10–12]. Creating a positive climate for individuals across all demographic groups and employment categories is critical to achieving a more equitable and just workplace [13]. While a positive climate alone is worthwhile, research indicates such shifts in climate also increase the productivity and success of academic departments [14, 15].

Identifying current strengths and areas in need of improvement is a logical first step to enhancing workplace climate [16]. Across colleges and universities, the act of ‘taking the temperature’ of an institution, alongside demographic data, often is achieved via climate surveys [17, 18]. Most climate surveys are administered campus-wide [19, 20]. Thus, overall responses may not accurately reflect or inform the climate and dynamics of individual departments. Yet, academic departments serve as the administrative structure for a particular discipline [21], each with their own mission and challenges. This structure distinctly impacts the people working directly within an individual department. Climate assessment, intervention, and improvement thus may be more feasible, relevant, and impactful at the departmental level.

Despite this need, department-specific climate surveys are rare. We found no published literature on climate surveys at the department level (though many departments likely have conducted related work that remains unpublished). Whereas campus-wide climate surveys can be initiated and supported with resources at the institutional level, designing and disseminating department-specific surveys likely relies on self-organization among department members. Furthermore, campus climate surveys typically target a specific educational or career stage (e.g., undergraduate, faculty) and thus fail to capture variation in perceptions of climate [22, 23]. A climate survey at Princeton University, for instance, revealed that experiences of climate were strongly influenced by educational/career stage and gender balances [24]. Taken together, department-specific climate surveys with questions tailored to various employment categories (i.e., faculty, graduate students, and staff) are needed to comprehensively assess workplace climate and identify areas in need of improvement.

To address this gap and inform work in our own department, we—a group of faculty, graduate students, and postdoctoral associates (i.e., staff)—developed and administered a climate survey specific to our academic workplace, the Department of Zoology and Physiology at the University of Wyoming. Here, we report on the results of the survey, with specific emphasis on the differences in responses across three employment categories: faculty, graduate students, and staff. We recognize the implicit hierarchies associated with these employment categories; herein, categories are listed in alphabetical order. Further, we discuss how survey results inspired current and future initiatives to improve the climate of our department and university.

Aside from informing work in our department, the main objective was to detail the process used to design, administer, analyze, and act on the survey, thereby providing a framework for improving workplace climate for all employees. For instance, we highlight the utility of a mixed-methods approach, particularly the nuances revealed by open-ended comments that were not apparent in responses to closed (e.g., Likert-scale) questions alone. Perhaps most importantly, we provide the survey itself (S1 File). Service work of this type is challenging and laborious, and often requires time and energy that is not incorporated into job responsibilities—individuals either work more or trade-off productivity in other areas that are formally evaluated. By providing the survey, along with a detailed description of our overall process for quantifying metrics of departmental culture, we aim to streamline this type of work for other departments wanting to assess their workplace climate, detect areas in need of improvement, and develop concrete actions for change.

## Materials and methods

### Setting

Laramie, located in southeastern Wyoming, has a population of 31,659 people and hosts the state’s only public, four-year-degree-granting institution, the University of Wyoming. In fall 2021, when this study occurred, the university had 1,064 faculty and academic professionals, 8,869 undergraduate students, 2,610 graduate/professional students, and 1,820 non-faculty staff. Within this broader sample, the Department of Zoology and Physiology employed approximately 30 faculty, 75 graduate students, and 18–21 staff. Staff covered four subcategories: ∼4 administrative staff, ∼6–7 postdoctoral researchers, and ∼8–10 research/laboratory and teaching/program staff (i.e., research associates, research scientists, museum curator, laboratory technicians, laboratory and advising coordinators). Given the low number of staff in each subcategory, we combined survey responses from all staff members into one category to protect anonymity. We did not survey custodial/maintenance staff because they were administratively outside the oversight of our department.

### Inception of climate survey and related efforts

Department-level climate efforts began nearly a year before the development of the survey. The origins of this effort are recounted in S2 File for those interested in climate work but not yet ready to invest in a departmental climate survey. Indeed, the foundations detailed in S2 File not only informed our survey work, but instigated it.

### Composition of survey team

A team, comprised of faculty, graduate students, and research staff created the survey. Differing perspectives and past experiences informed the process as we designed, piloted, distributed, analyzed, and reported on the survey. Throughout the process, members of the group reported to faculty, graduate students, and staff, and we consistently solicited feedback to ensure each employment category was represented.

### Survey design

We reviewed climate surveys conducted at several institutions to identify questions that would help determine areas in which our department was succeeding and where improvements could be made. Specifically, we reviewed survey reports found online or provided from survey leads at the University of Colorado Boulder [25], University of Michigan [26], Montana State University [27], Kansas State University [28], University of New Hampshire [29], University of Wisconsin Madison [30], Princeton University [31], and the University of Wyoming [32].

We next identified five general categories corresponding to indices of workplace climate: (1) Mental Health and Well-Being, (2) Importance of Diversity, Equity, Inclusion, and Justice (hereafter, DEIJ), (3) Sense of Belonging, (4) Safety, and (5) Work-Life Balance. We then drafted questions and cross-referenced them with a subset of the reports that used validated questions from published survey instruments.

Multiple question types can increase the clarity of survey responses, ultimately providing researchers with greater insight on participant experiences [33, 34]. Thus, we employed a mixed-methods approach by developing both closed-response (e.g., Likert-scale) and open-ended (text-fill) questions. With this format, (1) general questions for all employment categories and (2) specific questions for each employment category were created. Category-specific questions were developed in consultation with representatives of each employee category and thus were uniquely relevant to either faculty, graduate students, or staff. For example, faculty questions included matters of tenure and promotion, student questions addressed advisor relationships, and staff questions covered opportunities for professional development and feeling valued by the department. Therefore, whereas general questions were directly comparable across employment categories, category-specific questions were not. Rather, category-specific questions were intended to reveal nuanced experiences of climate exclusive to each employment category.

We conducted an expert panel validation [35] whereby two referees with extensive experience in survey development reviewed drafted questions. From their feedback, we then revised and finalized questions accordingly. To further ensure that questions were interpreted correctly and enabled respondents to answer as intended, we performed a face validation [35] by recruiting individuals within the department from each of the three employment categories to pilot the draft survey (*n* = 15 individuals, ∼12% of the department). We received edits from 11 of the 15 individuals asked to pilot the survey, and revised questions based on feedback.

The final survey consisted of 79 questions in nine categories (Table 1). In addition to the five categories describing workplace climate, one category described demography and three sections encompassed specific questions for each employment category: faculty, graduate students, and staff. The complete survey can be found in S1 File.

**Table 1:** Description and number of questions for each section included in a department climate survey, administered to the Department of Zoology and Physiology at the University of Wyoming during the fall of 2021.

Section	Description	Questions
Demographic	This section posed questions about employment category (i.e., faculty, graduate student, or staff) and self-identification within demographic groups (e.g., gender, sexual orientation)	17
Mental health and well-being	Mental health includes emotional, psychological, and social well-being. It affects how we think, feel, and act. There is no consensus around a single definition of well-being, but there is general agreement that at minimum, well-being includes the presence of positive emotions (e.g., contentment, happiness), the absence of negative emotions (e.g., depression, anxiety), satisfaction with life, fulfillment, and positive functioning.	9
Importance of DEIJ	Diversity, equity, inclusion, and justice; this acronym is rising in use (compared with DEI) due to the growing recognition that justice is an essential component of DEI work.	9
Sense of belonging	Belonging is the feeling of security and support when there is a sense of acceptance, inclusion, and identity for a member of a certain group. It is when an individual can bring their authentic self to work.	7
Safety	The aim of occupational safety and health is the promotion and maintenance of the highest degree of physical, mental and social well-being of workers in all occupations.	7
Work-life balance	A healthy work-life balance means feeling fulfilled and content in both the work and leisure realms of an individual’s life.	7
Faculty	Non-tenure and tenure track faculty	7
Graduate Students	Master’s and PhD track students	6
Staff	Administrative staff, research/laboratory staff, teaching/program staff, and postdoctoral researchers	10

*Notes*: The survey included a mixture of closed-response (Likert-scale) and open-ended (text-fill) questions.

We prioritized participation and candid responses to climate-related questions. Therefore, the entire survey was anonymous, and we took extra care to anonymize potentially identifiable responses. We also deliberately limited our access to demographic data by asking dichotomous questions, not identity-specific questions (S1 File). For example, rather than prompting respondents to self-identify with a specific gender, the question read, “*Do you identify as belonging to a gender that is currently or has been historically underrepresented in the fields of Science*, *Technology*, *Engineering*, *and Mathematics (STEM)*?”.

The climate survey group signed a confidentiality agreement to maximize buy-in and approval of the department (S1 File). Furthermore, we received written consent from participants via the consent section of the survey. This section of the survey was the first section with which respondents interacted. If participants did not provide consent, the survey automatically closed. We likewise required all respondents to confirm that they were age 18 or older. We submitted a full set of protocols, the full survey, and a description of purpose for the survey to the University of Wyoming Institutional Review Board (IRB). Based on the submitted protocols, UW IRB waived the need for consent and determined the survey was primarily an assessment, and thus did not require further IRB review nor approval.

We used Qualtrics© (Provo, UT) [36] to deploy the survey for three weeks during fall 2021. After the first email to the department, we sent weekly reminders to complete the survey. We provided a document with the survey that outlined the context, goals, format, confidentiality and anonymity, informed consent, important definitions and acronyms, and expected outcomes of the survey for respondents to reference while completing the survey (S3 File). Respondents were incentivized to participate by opting into the drawing of a $25 gift card (10 available with five guaranteed to graduate students). Gift cards were purchased with the department chair’s discretionary funds.

### Statistical analysis

For survey topics that consisted of a single question, we report the percent of respondents in each employee category that selected ‘yes’, ‘agree’, or ‘strongly agree’. Most survey topics, by contrast, consisted of several individual questions (S1 File). Given the similarity of questions within the same topic, fewer underlying constructs may better explain the observed responses. We therefore conducted factor analyses for these topics to reduce the complexity of responses by aggregating multiple questions into one or more indices [37]. We ultimately conducted factor analyses for questions in three of the five workplace climate categories, including importance of DEIJ, sense of belonging, and work-life balance.

To perform factor analysis, we first assessed correlation matrices for each topic to determine the number of factors to extract. Using these factor numbers, we conducted minimum residual factor analyses using the *fa*() function within the ‘psych’ package [38] in Program R version 4.1.1 [39]. Factors were rotated to an oblique solution using the *oblimin*() function from the ‘GPArotation’ package [40]. To ensure that we used the appropriate number of dimensions to represent the correlation matrix, we continued extracting factors until the chi-square of the residual matrix was not significant. We next obtained factor scores from each analysis using the *factor*.*scores*() function within the ‘psych’ package [41]. We then evaluated the standardized loadings of factor scores (based on the correlation matrix) to determine which individual questions from each topic belonged within the same index. Finally, we averaged responses within each index to obtain mean scores for each aggregate grouping of individual questions. For complete results of our exploratory factor analyses, including factor loadings on each factor score, please refer to S4 File.

Using the mean scores obtained from factor analyses, we conducted one-way Analysis of Variance (ANOVA) tests to determine if responses were statistically different among faculty, graduate students, and staff. One-way ANOVAs were performed using the *aov*() function in Program R, with Tukey Honest Significant Difference tests computed (using the *TukeyHSD*() function) to identify which groups were statistically different. Prior to analysis, we tested for violations of ANOVA assumptions, including normality (using the *shapiro*.*test*() function in the ‘stats’ package) and homogeneity of variances (using the *leveneTest*() function in the ‘car’ package [42]). We performed Kruskal-Wallis rank sum tests using the *kruskal*.*test*() function within the ‘stats’ package if the assumption of normality was violated.

### Emergent thematic coding of open-ended responses

Two investigator pairs from the survey team independently coded the open-ended components of each category; doing so, the coders began to identify shared themes and unique situations in the experiences reported by survey respondents [43]. Each coder recorded possible emergent themes. These possible themes were then standardized between coding pairs to define an intial, common slate of codes. A second cycle of coding, conducted independently by two members of the team, refined the initial slate of codes into three themes based on recommended actions (hereafter action themes) that were consistent across the five question categories (e.g., safety, sense of belonging). This development process allowed for consensus-building between coders, resulting in a coherent, robust representation of the emergent themes present in the responses [44, 45].

Three action themes were ultimately identified: equity, community, and accountability. Each open-ended response was then coded into these themes. Two co-authors conducted independent coding, each doing 50% of the responses. A third co-author coded a 30% overlap of the whole set of responses to assess intercoder reliability. Specifically, we assessed intercoder reliability using the *kappa2*() function within the ‘irr’ package [46] in Program R to calculate a Kappa coefficient of agreement among coders for each theme [47]. Kappa values indicated intercoder agreement above 95% for all three themes, establishing sufficient reliability between coders [48]. In the few instances wherein there was disagreement about how a response should be coded, another co-author was asked to break the tie [49]. Each response was coded for inclusion in each of the themes with which it aligned. As a result, some responses were coded into one, two, or even three themes. We do not report any direct quotes from responses to protect anonymity.

## Results

### Demographics

The departmental climate survey achieved a total response rate of 82%. Of the 120 individuals who completed the survey (out of a possible 147), 26 self-identified as faculty, 67 as graduate students, 16 as staff members, and 11 elected not to specify their employment category. Fifty-nine of 109 respondents (54%) self-identified as a gender underrepresented in STEM, and 54 (50%) had a primary caregiver with a graduate or professional degree. Participants reported much lower rates of self-identification with other identities underrepresented in STEM: race/ethnicity (6%), sexual orientation (3%), socioeconomic (2.5%) and disability status (4%).

### Mental health and well-being—closed-responses

**Fig 1:**
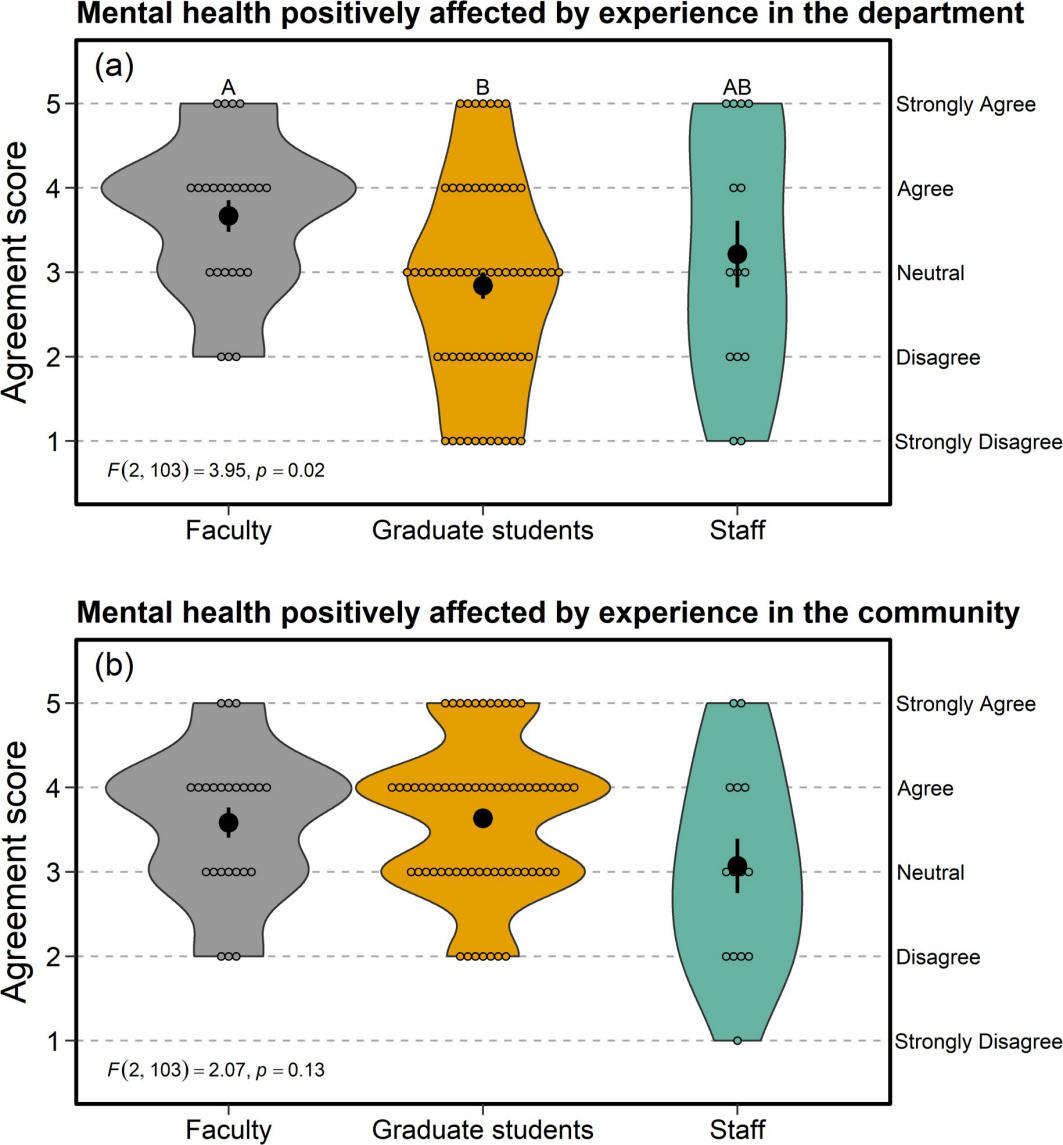
Compared with faculty members, graduate students in the Department of Zoology & Physiology at the University of Wyoming (a) agreed to a significantly lesser extent that their mental health has been affected positively by their experience within the department. By contrast, graduate students tended to agree that (b) their mental health has been affected positively by their experience within the Laramie community. Agreement scores on the y-axis denote survey responses to questions within the Mental Health and Well-Being section. Larger, filled circles with error bars indicate mean ± SE, whereas smaller, open circles display the raw data. Violin plots (grey, orange, and green polygons) display the full distribution of the raw data, including the density of each variable. Thus, the different widths in the x-dimension of each polygon are simply peaks in the data distribution and indicate the number of respondents with that particular score.

Faculty tended to agree that their mental health had been affected positively by their experience within the department, whereas staff and graduate students were more neutral, with graduate student agreement significantly lower than faculty (*F*_2, 103_ = 3.95, *p* = 0.02; Fig 1A). By contrast, both faculty and graduate students tended to agree that their mental health had been affected positively by their experience within the Laramie community, with staff responses closer to neutral, and no statistical differences among groups (*F*_2, 103_ = 2.07, *p* = 0.13; Fig 1B).

### Importance of DEIJ—closed-responses

Eighteen percent of respondents felt discriminated against in the department workplace when grouping ‘yes’ responses across ‘sometimes’, ‘often’, and ‘very often’. Specifically, 19% of faculty, 10% of graduate students, and 21% of staff felt discriminated against according to this grouping. Further, 50% of the participants who did not identify their employment category felt discriminated against, which may have influenced their decision not to identify.

**Fig 2:**
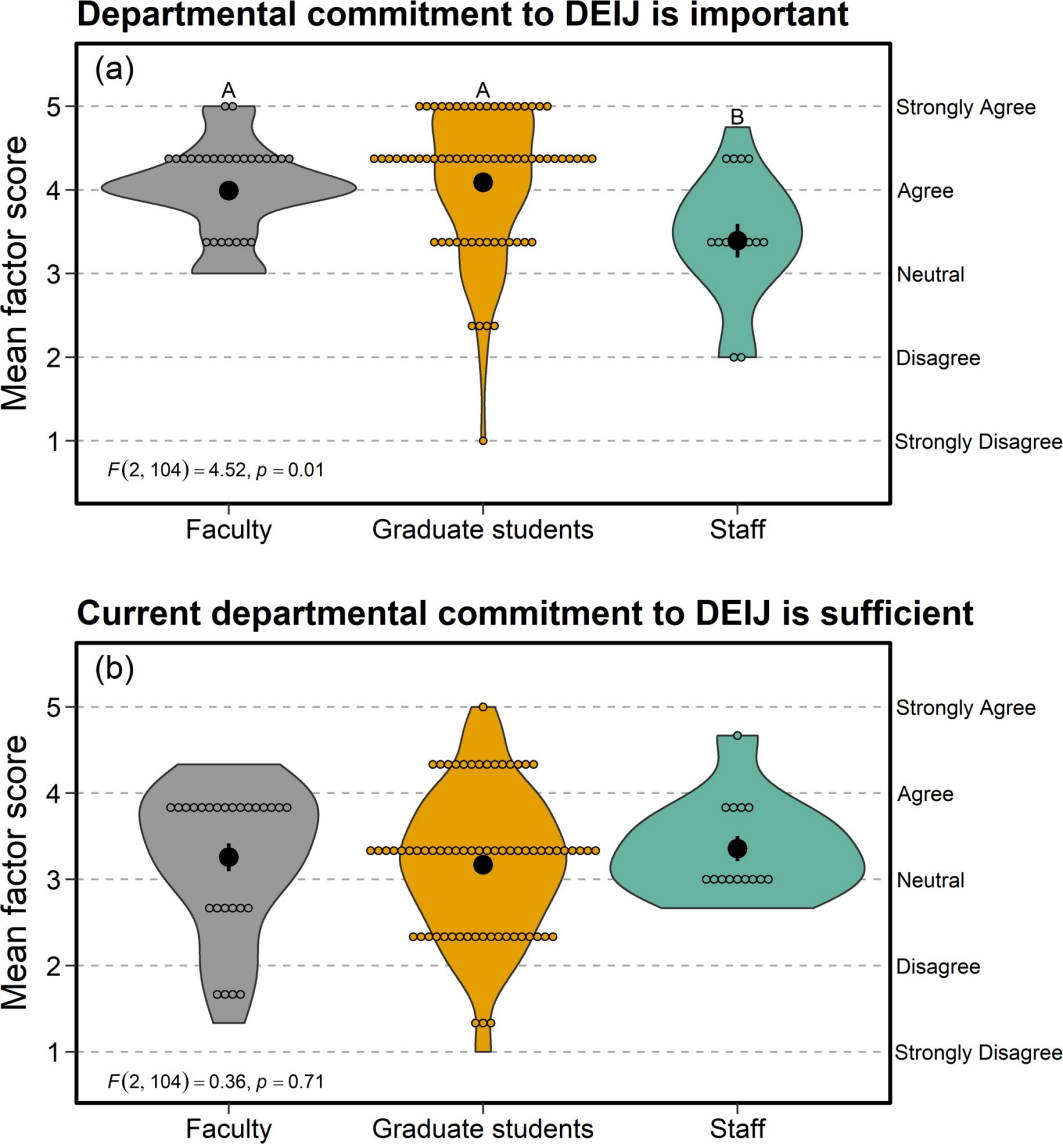
Although staff members in the Department of Zoology & Physiology at the University of Wyoming felt that (a) institutional commitment to DEIJ was less important than did faculty and graduate students, none of the employment categories (b) agreed that the current departmental commitment to DEIJ-related issues is sufficient. Mean scores on the y-axis represent indices derived from factor analysis of responses within the Importance of DEIJ section. Larger, filled circles with error bars indicate mean ± SE, whereas smaller, open circles display the raw data.

Factor analysis aggregated survey responses within the Importance of DEIJ category into three indices (S4 File). We did not discover any violations of ANOVA assumptions in the mean scores obtained from factor analyses (e.g., Shapiro-Wilk test for normality, *p* > 0.05; Levene’s test for homogeneity of variances, *p* > 0.05). Faculty and graduate students agreed that departmental/institutional commitment to DEIJ-related issues is important, whereas staff reported more neutral and significantly lower responses (*F*_2, 104_ = 4.52, *p* = 0.01; Fig 2A). However, none of the employment categories agreed that the current departmental commitment to DEIJ-related issues is sufficient; all groups reported neutral responses to this question (mean score across groups = 3.21, SE = 0.08) with no statistical differences among employment types (*F*_2, 104_ = 0.36, *p* = 0.70; Fig 2B).

### Sense of belonging—closed-responses

**Fig 3:**
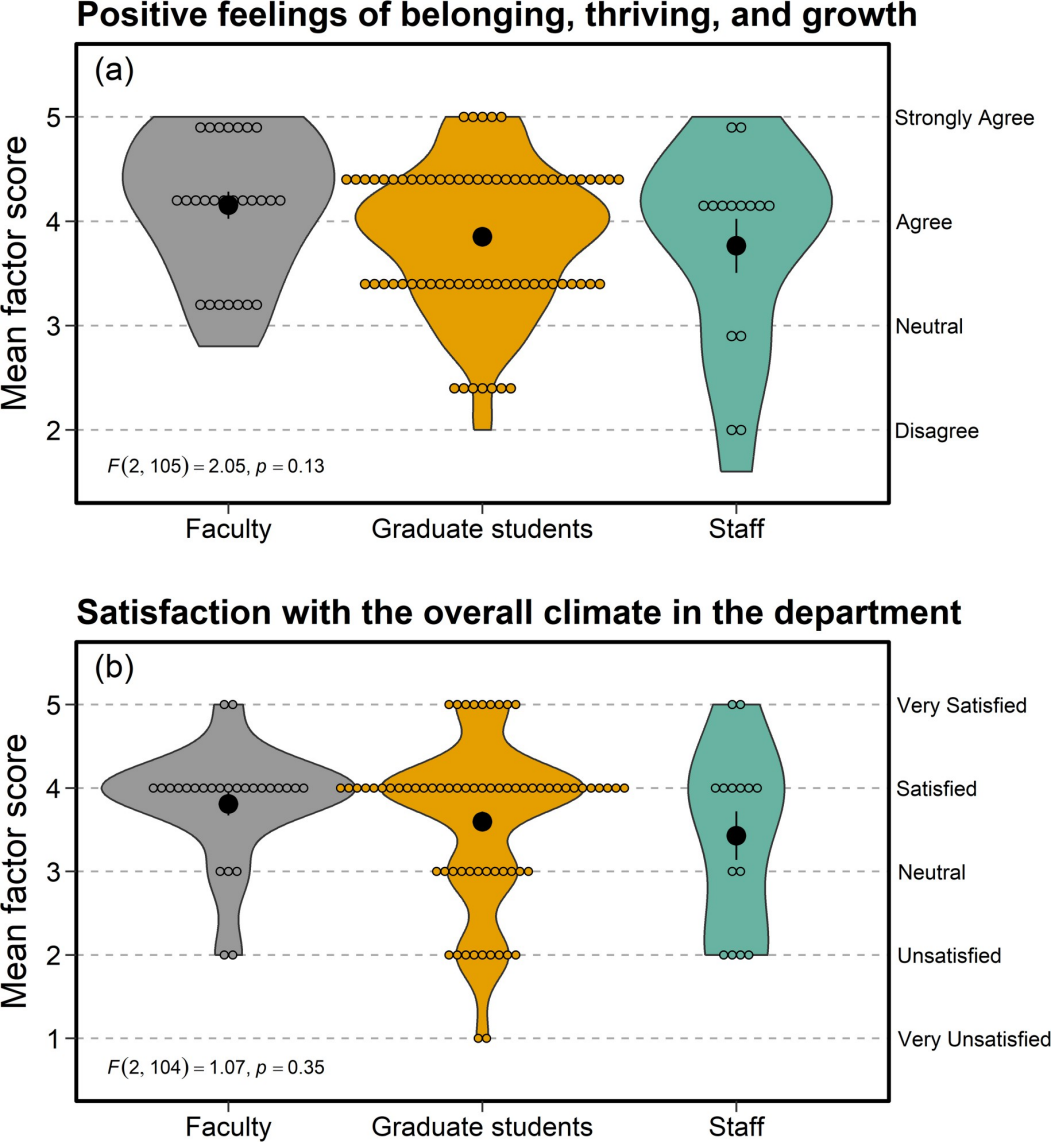
Faculty, graduate students, and staff in the Department of Zoology & Physiology at the University of Wyoming generally reported (a) positive feelings of belonging and (b) satisfaction with the current departmental climate. Mean scores on the y-axis represent indices derived from factor analysis of responses within the Sense of Belonging section on the departmental climate survey. Larger, filled circles with error bars indicate mean ± SE, whereas smaller, open circles display the raw data.

Factor analysis aggregated survey responses within the Sense of Belonging category into six indices (S4 File). We did not discover any violations of ANOVA assumptions in the mean scores obtained from factor analyses (e.g., Shapiro-Wilk test for normality, *p* > 0.05; Levene’s test for homogeneity of variances, *p* > 0.05). Faculty, graduate students, and staff all agreed that they felt valued by their peers (mean agreement score across groups = 4.22, SE = 0.05), supervisors (mean score = 4.02, SE = 0.11), and employees/students (mean score = 4.19, SE = 0.09), with no statistical differences among groups (peers: *F*_2, 103_ = 0.77, *p* = 0.47; supervisors: *F*_2, 103_ = 0.98, *p* = 0.38; employees/students: *F*_2, 103_ = 2.64, *p* = 0.08). However, staff agreed to a lesser extent on overall positive feelings of belonging (Fig 3A) and satisfaction with current departmental climate (Fig 3B). Mean responses of staff were closer to neutral compared with responses from faculty and graduate students, though there were no statistical differences among employment categories for either metric (Fig 3A and 3B).

### Safety—closed-responses

**Fig 4:**
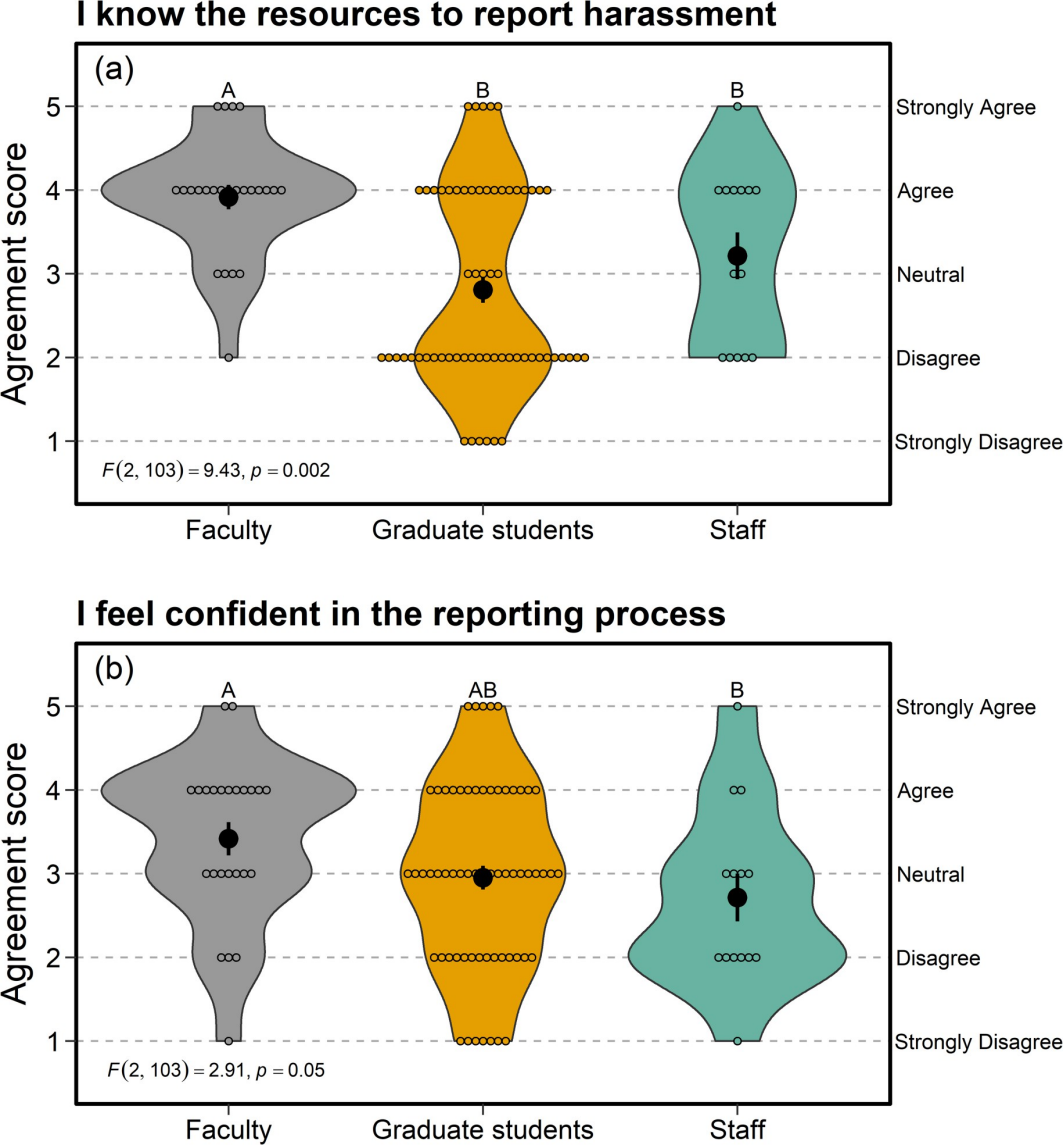
Compared with graduate students and staff members, faculty in the Department of Zoology & Physiology at the University of Wyoming agreed to a greater extent that they were aware of the resources to report harassment (a) and were confident in the reporting process (b). Agreement scores on the y-axis denote survey responses to questions within the Safety section of the climate survey. Larger, filled circles with error bars indicate mean ± SE, whereas smaller, open circles display the raw data.

Faculty agreed that they were aware of the resources to report harassment (Fig 4A) and were confident in the reporting process (Fig 4B). By contrast, graduate students and staff agreed to a lesser extent with both statements above, with mean responses closer to neutral (Fig 4A and 4B). Similarly, compared with graduate students and staff, faculty agreed to a greater extent that they were comfortable reporting harassment if they experienced it (*F*_2, 103_ = 4.55, *p* = 0.01) or witnessed it, though results were not significantly different among employment categories for reporting harassment if witnessed (*F*_2, 103_ = 2.39, *p* = 0.11).

### Work-life balance—closed-responses

Staff and graduate students generally felt that neither their supervisor nor their peers expected them to work over 40 hours per week. A larger percentage of faculty, by contrast, were either unsure (21% of responses for supervisor expectations, 23% for peer expectations) or reported explicit (6% supervisor, 3% peer) or implicit (36% supervisor, 27% peer) expectations to work more than 40 hours per week. Fewer staff (31%) agreed that having a dependent to care for would negatively impact their career, compared with 50% and 54% for faculty and graduate students, respectively. However, a similar percentage of faculty (46%), staff (43%), and graduate students (48%) reported that if they had a child, they would feel pressured to return to work before they wanted. Almost all faculty and staff (96% and 100%, respectively) reported that personal relationships (e.g., family, partner) are important in shaping their career decisions, compared with only 70% of graduate students.

Factor analysis aggregated some of the survey responses within the Work-Life Balance category into three indices (S4 File). We did not discover any violations of ANOVA assumptions in the mean scores obtained from factor analyses (e.g., Shapiro-Wilk test for normality, *p* > 0.05; Levene’s test for homogeneity of variances, *p* > 0.05). Faculty, graduate students, and staff all agreed (mean agreement score across groups = 3.88, SE = 0.09) that they were expected to take time off for holidays, illness, and family matters, with no statistical difference among groups (*F*_2, 104_ = 2.27, *p* = 0.11). However, all groups were neutral regarding clear communication of procedures for taking time off (mean agreement score across groups = 2.92, SE = 0.12), again with no differences across groups (*F*_2, 103_ = 1.69, *p* = 0.19).

### Employee category-specific questions

Of the 26 respondents who identified as faculty and 67 who identified as graduate students, all completed the category-specific questions. Of the 16 respondents who identified as staff, only nine answered the staff-specific questions. Here we present results for a subset of the category-specific questions that were most relevant to each employment group; the results for the remaining questions can be found in S5 File. As stated above in the Materials and Methods section, category-specific questions were unique to each employment group (e.g., faculty were asked about tenure and promotion), such that these questions are not comparable across categories.

**Fig 5:**
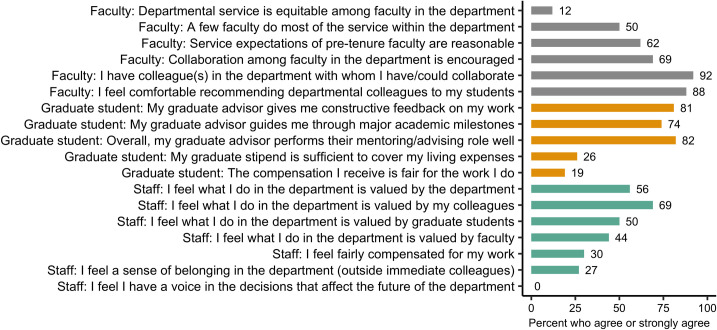
Percent of faculty (grey filled bars), graduate students (gold filled bars), and staff (teal filled bars) who agreed or strongly agreed with employee category-specific questions. Category-specific questions were developed in consultation with representatives of each category and thus were uniquely relevant to either faculty, graduate students, or staff. The figure presents a subset of the category-specific questions deemed most relevant to each employment group (results for the remaining questions can be found in S5 File).

Faculty generally agreed that collaboration within the department was strong (Fig 5). Faculty also generally agreed that department service expectations were reasonable; however, faculty believed that a few faculty did most of the service and that service was not equitable within the department (Fig 5). Graduate students generally agreed that their graduate advisors did a good job with overall mentorship, specifically by providing constructive feedback and guiding them through academic milestones (Fig 5). Graduate students did not think that their stipend was sufficient to cover living expenses, or that financial compensation was fair for the work that they did (Fig 5). Generally, staff felt valued by their colleagues, but less by the department, faculty, and graduate students (Fig 5). Staff generally felt that they were not compensated fairly for their work, and that they did not have a voice in decisions within the department (Fig 5). This section also included open-response questions. However, to preserve anonymity of respondents, we only report aggregate results of open-response questions, which can be found in S5 File.

### Emergent thematic coding of open-ended responses

Of the 120 individuals who completed the survey, 78 provided at least one open-ended comment (65% of respondents). The emergent themes of equity, community, and accountability were common across all employment categories and survey sections (e.g., safety, sense of belonging), though the frequency of each theme varied: equity (76 instances), community (54), and accountability (32). The closed-response results had mean agreement scores generally between 3 and 4 (neutral to agree) for statements reflecting a positive workplace climate (Figs 1–4); however, open-ended responses revealed both positive and negative perspectives on workplace climate (Table 2). Whereas some responses to Likert-type questions picked up on these variations, the nuance and range of experiences were not apparent without including the open-responses. We do not report verbatim quotes from open-ended comments to protect the anonymity of respondents. Rather, in the Discussion section below, we review how these comments helped to identify concrete actions that can improve the climate of our department.

**Table 2:** Topics mentioned in open-ended responses revealed a more nuanced department climate than would be apparent from closed responses alone. Example topics illustrate how comments were coded into the three emergent themes of equity, community, and accountability.

Survey Section	Equity	Community	Accountability
Mental health and well-being	Lack of mental health care benefits for students and staff	Reports of mental health discussions being normalized	Experiences with toxic supervisors and expectations of workaholism
Importance of DEIJ	Appreciation and criticism of existing DEIJ department efforts	Concerns about lack of visible diversity in the department	Experiences with power imbalances between supervisors and students
Sense of Belonging	Staff excluded from decision-making processes	Appreciation of and desire for more activities to build department community	Explicit policies that are communicated cohesively to all employee categories
Safety	Student concerns regarding medical costs and inadequate insurance	Desire to co-produce department handbook that includes a safety manual	Requests for field and safety trainings and protocols
Work-life balance	Perceptions that work-life balance in the department is better than other institutions	Experiences of peers supporting healthy work-life balance	Lack of clear protocols for taking time off

## Discussion

We developed and administered a climate survey for faculty, graduate students, and staff within the Department of Zoology and Physiology at the University of Wyoming (UW) during fall 2021. We employed a mixed-methods approach, with a combination of (1) general questions for all employment categories, (2) specific questions for each employment category, and (3) open-ended responses. The response rate of 82% aligns with research indicating that valid response rates can range from 60–100% [50]. Mean agreement scores for general statements in alignment with positive workplace climate were 3–4 (neutral to positive) overall. However, faculty generally reported higher agreement with these positive aspects of workplace climate than did staff, and graduate students often fell in the middle of these two groups. The only mean responses below neutral for these general statements were for graduate students with regard to mental health and for graduate students and staff with regard to harassment reporting. Category-specific and open-ended responses were less indicative of a positive workplace climate, including reports of extremely negative and concerning experiences. Three common themes for recommended action emerged from open-ended questions that helped identify areas in need of improvement: equity, accountability, and community. Below we discuss how these themes encompass the five sections of the survey and inform current and future efforts in the department.

### Mental health and well-being

Negative effects on the mental health of graduate students from experiences within our department (e.g., Fig 1A, open-ended responses) are consistent with the pervasive mental health crisis among graduate students worldwide [51, 52]. Results in this section helped to motivate the formation of the Mental Health and Wellness Group, which is now recognized as a formal committee within our department. This committee supports and builds community among department members, provides information on mental health services on campus and in the community, manages and maintains a food pantry, and catalyzes related discussions internally and with invited speakers.

Many open-ended responses in this section mentioned equity, particularly with respect to graduate student pay, another widespread issue in academia that influences mental health and well-being [53, 54]. Such recurring anecdotes of financial insecurity inspired a group of department faculty to advocate for increasing the stipends of graduate students across the university. University-funded stipends increased by 4% in 2022 [55]. Yet, this increase does not meet living wage needs in Laramie, and thus the department has formalized a committee to identify department-level methods that could provide more equitable pay and benefits for graduate students. Similarly, few staff felt that they are fairly compensated for their work (Fig 5). UW faculty and staff received $1,400–1,900 base pay raises in 2022 [55]. However, the raise does not address all reported concerns, as some staff reported that their health benefits have been cut repeatedly. Our department does not have control over staff salaries and benefits. A department group advocating for increasing staff pay and improved benefits across the university may therefore be vital for staff retention.

### Importance of DEIJ

Most employees agreed that departmental commitment to DEIJ is important (e.g., Fig 2A). Open-ended responses in this section commonly mentioned equity, often in reference to educating department members on anti-racism, anti-sexism, and other DEIJ topics (suggestions included workshops, trainings, and course offerings). Based in part on these results, the DEIJ reading group (see S2 File) continues each semester, with a member of the previous group leading the next cohort. Each semester’s cohort adopts a concrete action item to improve our department. Such actions have included (1) an extensive spreadsheet of more diverse speakers possibly available for seminars, (2) efforts to establish a mentorship support system for faculty and graduate students, and (3) a database of resources to better integrate the work of individuals from underrepresented and marginalized minority groups into course curricula. Further, other departments across our campus are interested in conducting DEIJ and climate work like ours, several of which have installed readings groups and recurring courses modeled in part after our initial efforts. We will continue to collaborate with external departments and consult on their climate work.

Although these are encouraging steps forward, DEIJ-related actions are often difficult to sustain on a long-term basis because of structural biases within academia. DEIJ-related work is typically not incentivized or compensated. Thus, mitigating equity issues requires employees to devote additional time and energy that is not incorporated into their job descriptions. This inequity in DEIJ-related service is a common theme within higher education, with disproportionate labor performed by underrepresented and contingent academics [56, 57]. Furthermore, most faculty in our department believe that service is already not equitable among faculty members (Fig 5). Indeed, open-ended responses called for placing more value on service in tenure and promotion. Rewarding service, including DEIJ engagement, may help engender a commitment to DEIJ among applicants and employees, ultimately reinforcing DEIJ principles in the hiring and promotion of employees within our department [58].

### Sense of belonging

The Sense of belonging section provides an excellent example of the discrepancy we observed between responses to the general versus employee category-specific questions. For instance, whereas staff responses were not overwhelmingly negative in the general questions for this section (e.g., Fig 3), responses to the staff-specific questions revealed that most staff do not feel a sense of belonging in the department (S5 File). Recommendations to increase sense of belonging from open-ended responses often mentioned community (one of the three recommended-action themes), with particular emphasis on cluster hiring, cohort recruitment, and departmental events to foster support and inclusion among peer groups. Partially in response to these results, two postdocs within the department created a cohort-building course for first-year master’s students, entitled Topics and Discussions in Ecology. The course covers advanced fundamentals in ecology and aims to create a sense of community within master’s-level cohorts (such a course already exists for PhD students in ecology). While such cohort building is excellent for zoology and ecology students, a divide between zoology and physiology remains in the department; this divide was noted frequently in the open-ended responses.

The Mental Health and Wellness Group initiates shared activities for all department members, though other efforts are warranted to help build a sense of community and collaboration between employees in the zoology and physiology disciplines. Furthermore, while such efforts are a start, we also recognize that social activity outside work hours may be counter-productive. Adding commitments may be differentially challenging for different groups, thereby reinforcing divisions that impede general feelings of belonging. A more productive shift may be to follow-up on staff suggestions to audit department operations and identify where staff can be included in decision-making and hiring processes. The department also could establish a regular check-in system whereby staff can gather and provide feedback to the department.

Cluster hiring, as mentioned in several open-ended responses, may help foster interdisciplinary collaboration [59], though evaluations of such practices have produced mixed results [60]. Cluster hiring also can increase representation from underrepresented minority groups, as well as mitigate the isolation often felt by said individuals [61]. However, hiring people with diverse backgrounds (at faculty, graduate student, or staff levels) without regard for the organizational mechanisms that support or impede professional success can lead to a ‘bait and switch’ whereby individuals enter a negative workplace climate [62]. Successful cluster hiring therefore requires financial investment, informed outreach, and rigorous adherence to inclusive, equitable hiring practices to recruit and retain diverse personnel, along with prior work to develop a climate that can genuinely support such hires.

### Safety

All question types revealed a general sense of uncertainty and lack of confidence in current safety procedures (e.g., reporting harassment, safety in the field, Fig 4), except for faculty members (perhaps because they are required to take Title IX training). Open-ended responses commonly mentioned accountability (the third recommended-action theme), which included a lack of transparency and clarity in department policies. Several responses called for a department handbook that details everything from reporting harassment to evaluating graduate student and supervisor performance. Such processes could help protect all employees, especially those in lower rank or status positions within the power structure [63]. Currently, the department’s Graduate Advisory Board admits graduate students and oversees their progress within our department, and the mentor-mentee resources developed by the DEIJ reading group aim to facilitate mutually beneficial advisor-student interactions and prevent toxic relationships. However, no formal mechanisms currently exist in our department to address such issues in advisor-student or supervisor-staff dynamics. A few open-ended responses suggested that an ombuds position could help mediate conflicts; this is a position that many other institutions and professional societies have adopted recently [64, 65]. Indeed, our university has established such an office since we conducted our survey, though it is focused primarily on undergraduate support. Thus, its capacity to mediate department-level issues remains to be seen.

Many open-ended responses requested that faculty formulate and implement safety protocols for laboratory and field work. Negative experiences (e.g., harassment, abuse, injury) in these settings can disrupt career trajectories [66] and disproportionately affect underrepresented minorities, often dissuading them from continuing in their jobs or career tracks [67–69]. Clear safety protocols and risk management plans can create a more equitable code of conduct through addressing injury, harassment, discrimination, and bullying. Preventing and dealing with such issues may help retain employees from diverse groups in the disciplines of zoology and physiology [70, 71]. Finally, we did not include laboratory and field technicians in our survey because these positions are seasonal/temporary and most such employees were no longer with the department. Future efforts to survey temporary technicians about their experiences likely would better inform the department on the culture and climate experienced by employees within field and laboratory settings.

### Work-life balance

All employment categories agreed that they were expected to take time off, but that procedures for taking time off are unclear. Open-ended responses commonly mentioned accountability, specifically with respect to clarity in department policies for requesting and taking time off. Setting transparent protocols for taking time off may help address the sentiment of many department members that, if they had a child, they would feel pressured to return to work before they wanted (S5 File). Academic norms often disproportionately penalize women for family responsibilities [72, 73], such that clear family-friendly policies may result in improved gender equity in our department [74]. Such shifts may be particularly important for graduate students and staff, given faculty responses indicated high satisfaction with work-life balance and few concerns about caregiving constraints. Further, detailed policies and protocols appeared as a common thread throughout each of the five sections above, suggesting that clarity and accountability would likely help promote an overall positive climate. Finally, we acknowledge that much of work-life balance is outside of the scope of influence of the department, as employees have various life challenges and commitments to family, the community, and beyond. However, clear protocols for time off, remote work, and flextime may ensure that employees can take the time they need to handle challenges and enjoy activities that are essential to their overall well-being.

### Caveats

A lack of significant differences across employment categories in several general questions may have resulted from experiences of climate being influenced more by social identities (e.g., gender, race) than employment category. For instance, gender-based harassment and discrimination may play a larger role in individual experiences of climate, irrespective of employment category [12]. Unfortunately, having fewer participants in department-specific surveys (versus campus-wide) limits the statistical power necessary to evaluate differences in climate experience across social identities (e.g., race, religion, disability status). Further, low statistical power in departmental surveys affirms the need to include open-ended responses, as anecdotes may be more indicative of workplace culture than statistical analyses with low sample sizes. That said, we acknowledge that open-ended prompts can insert bias as individuals with extreme experiences may be more likely to report [75]. We did not resist this potential bias in reporting, however, because our departmental culture is only as strong as the most negative experience, and negative reports clarified areas in need of improvement. Also, approximately two-thirds of participants provided open-ended comments (78 of 120), suggesting that reporting bias may not have been a significant issue in our study.

Low sample sizes were particularly evident in staff. We grouped administrative and academic staff together in analyses to protect anonymity; however, this aggregation over-generalized the experiences of climate in each staff category. For instance, staff members have vastly different job descriptions and work expectations depending on their position. Thus, future climate work could consider developing a separate survey to better capture the nuance and issues that are important to staff well-being and success. Furthermore, staff respondents generally felt that they did not have much say in the department (S5 File). That sentiment was reinforced by the fact that very few respondents’ recommended actions (and current department initiatives) address staff needs. Departmental investment is needed to better engage staff and facilitate their inclusion in activities and decisions. Such efforts may help to better target staff needs and enhance their sense of belonging.

We acknowledge potential biases and limitations in our factor analyses. Factor analysis alone does not reveal the cause of covariability, such that naming factors can be problematic and reliant on interpretation from researchers [76]. Further, variables may correlate with each other to produce a factor despite having little underlying meaning for the factor (i.e., correlation between unrelated variables). Grouping unrelated variables together can result in a loss of information and spurious results [76]. Our factor analyses included questions from within the same subject category, however, so we were confident that the analyses were detecting appropriate underlying structures in the data (S4 File).

We also acknowledge potential biases stemming from the lack of racial and ethnic diversity within our survey development team (most team members identify as White; capitalized per [77]). Our unconscious biases could have ranged from the types of questions developed to our interpretation of the responses [78]. Survey results may contain similar biases; only seven of 109 survey participants identified as belonging to a racial or ethnic group that is currently and/or historically underrepresented in STEM fields, suggesting our results and subsequent initiatives may not be generalizable to other departments with greater ethnic diversity. However, the survey approach (and associated actions) we describe is one of many steps that academic departments can take to enhance climate as a critical part of attracting and retaining diverse new members.

Finally, in the open-ended responses, a few individuals acknowledged that the COVID-19 pandemic influenced their employment experience, particularly with respect to isolation and emotional wellness [79]. Time spent in one’s position and/or time in the department also could have biased our results. Recent hires have less experience with workplace culture and may lean toward more neutral responses. Analyses that include time in the department would help quantify the relative contribution of employment duration to experiences of workplace climate. Further, anonymizing the survey participants precludes our ability to track responses of the same individuals through time. Still, our department is committed to administering the climate survey regularly (every three years) to evaluate whether departmental initiatives are improving overall experiences of workplace climate.

## Conclusions

Multiple question types helped reveal nuance regarding the climate of our department. Whereas many employees reported neutral experiences of workplace climate in the general questions, open-ended responses uncovered both positive and negative experiences that would have gone undetected using closed-response questions alone. Notably, open-ended responses identified concrete action items for improving departmental climate, some of which have been implemented already (e.g., cohort-building courses, stipend increases for graduate students) while others constitute future initiatives (e.g., department-level ombuds, mentoring workshops for faculty). Further, specific questions to faculty, graduate students, and staff facilitated the acquisition of nuanced experiences of climate that were unique to each employment category. Importantly, survey results provided quantitative support for areas in need of improvement, and recommended actions point us toward tangible, achievable progress. These outcomes have heartened our department—organizational change can seem daunting, but real, meaningful change is possible even at the department level.

**Fig 6:**
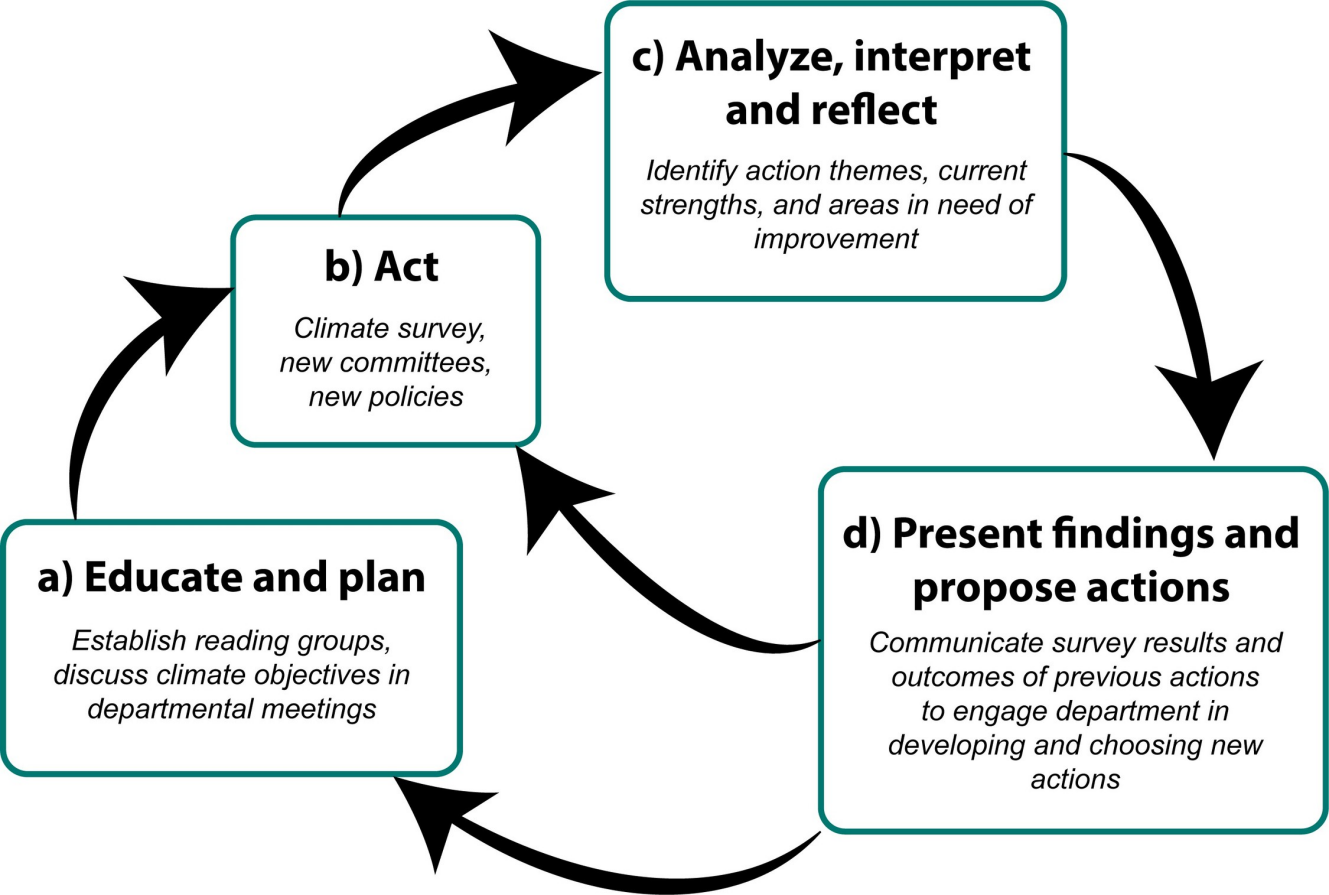
Conceptual diagram of our overall process to evaluate and improve workplace climate. Bold lettering indicates procedural steps (themes) and italics denote actions conducted within each step. (a) Educate and plan. When beginning climate work, start by educating department members about issues related to mental health and well-being, diversity, equity, inclusion, and justice, sense of belonging, safety, and work-life balance. For instance, our department formed a reading group, wherein members worked in small groups to learn about potential actions that could make our department a better place (see S2 File). (b) Act. Next, commit to an action item. Our department administered a climate survey to quantify current strengths and areas in need of improvement. (c) Analyze, interpret, and reflect. Then, analyze and interpret the data and results of the action item. What was learned from this process? (d) Present findings and propose actions. Communicate major findings to the department and co-develop targeted goals and future actions. Importantly, this process is iterative and cyclical, with each step informing the next via feedback loops. Allowing each step to guide the next can help the department remain engaged in climate work by consistently evaluating and improving its workplace environment.

More broadly, we provide a general overview of our process to evaluate and improve departmental climate (Fig 6). We hope that sharing our methods and experience will enable other departments to save time and effort by adopting our resources and modifying them to their specific needs. Together, we can inform broader efforts to achieve a positive work environment for all employees.

## Supporting information

S1 File Complete climate survey administered to the Zoology & Physiology department. (DOCX)

S2 File Details regarding the inception of our climate survey and related efforts. (DOCX)

S3 File Context, consent, definitions, and acronyms for the 2021 climate survey. (DOCX)

S4 File Complete results from the exploratory factor analysis. (DOCX)

S5 File Complete results from the employee category-specific questions. (DOCX)
