# Supplementary material for: Equity, community, and accountability: Leveraging a department-level climate survey as a tool for action
Source: PLoS One. 2023 Aug 17;18(8):e0290065. doi: 10.1371/journal.pone.0290065 (PMC10434968; doi:10.1371/journal.pone.0290065)
Supplement: S1 File — (DOCX) [file pone.0290065.s001.docx]

**Supplement 1**

**Journal Name:** *PLOS ONE*

**Manuscript Title**: Equity, community, and accountability: leveraging a department-level climate survey as a tool for action

**List of Authors:**

Gabriel M. Barrile, Riley F. Bernard, Rebecca C. Wilcox, Justine A. Becker, Michael E. Dillon, Rebecca R. Thomas-Kuzilik, Sara P. Bombaci, and Bethann Garramon Merkle

Corresponding Author Email: [gbarrile15@gmail.com](mailto:gbarrile15@gmail.com)

Supplement 1 – Zoology & Physiology Climate Survey

**Confidentiality agreement | Department of Zoology & Physiology Climate Survey**

As a member of the JEDI Core Group in the Department of Zoology & Physiology at the University of Wyoming, I may have access to sensitive or confidential information. This confidentiality agreement serves to verify that I have been made aware of the strict prohibition against inappropriate use of sensitive or confidential information. I understand that all members of the Department of Zoology & Physiology expect me to hold in confidence any information I may become privy to in the course of my work. Because this information is solely available to me as a result of my involvement with the JEDI Core Group, I will not discuss, use, forward, print, copy, save data to unsecure/personal locations, photograph, record or otherwise disseminate any confidential or sensitive information that is given, shown, or available to me, or which otherwise comes to my attention, for purposes outside the legitimate scope of my work. I further agree that during the term of my association and following my separation with such association, I will be bound by this agreement. I am aware that failure to abide by this agreement may subject me to disciplinary action up to and including my immediate exclusion from this position.

The above letter was signed by all members of the survey team.

NOTE: This is a plain-text export from Qualtrics. Formatting within a survey system provides a more legible and readily navigable survey for respondents.

Zoology & Physiology Climate Survey

Introduction

Welcome to the Department of Zoology and Physiology Climate Survey. 
Before you begin, we've provided a context section for you to review. However, If you would like to refer to it after taking the survey, a PDF is available here:

***The overarching goal of this survey*** is to characterize departmental climate (e.g., the way that people experience being part of the department). 

Climate is a key determinant of diverse aspects of success in work settings, including in academia. It will be valuable for us all to understand this experience from the perspectives of students, staff, and faculty. 

By identifying areas of excellence as well as areas in need of improvement we can target specific future actions to build on our successes and tackle any issues that emerge. 

Regular administration of the survey provides a useful tool for tracking department climate over time and for measuring the effectiveness of actions we take to improve the department. Therefore, we plan to administer this survey on a 3-year rotation.

We recommend using Chrome web browser to participate in the survey. 

***Note:*** throughout the survey you'll see words in green, hover your mouse over these words to see a definition.

What would you like to do next?

- Continue reading context, goals, participants, informed consent, etc., sections of this introduction.
- Skip to confidentiality and anonymity clarifications.
- Skip to consent form and start taking survey. (Note: We think the context is important for everyone to know. If you would like to refer to it after taking the survey, please refer to the pdf above.)

*Display This Question:*

*If What would you like to do next? = Continue reading context, goals, participants, informed consent, etc., sections of this introduction.*

**Context**
This is an ideal time to implement this survey, given: (a) the department was externally reviewed in 2020, (b) our programs are currently under review as part of university-wide strategic planning, and (c) numerous recent efforts are underway by faculty, staff, and students to enhance our department. 

These efforts include strengthening the graduate program with a new charge for the Graduate Advisory Board and standardized processes, bolstering undergraduate research, jumpstarting fundraising, ongoing action-oriented justice, equity, diversity, and inclusion (JEDI) reading groups, and interest in enhancing community between disciplines and career levels. 

If anything, the current reorganization discussion makes this all the more pressing. We want to identify key aspects of the departmental climate we value and take those positive aspects of ZP into the future. We also intend to use “need to change” results to inform the future of ZP.

**Goals** 
This climate survey will inform all of these efforts and, critically, will be an ongoing process with results driving action. Subsequent surveys (every 3 years) will evaluate the effectiveness of actions, track climate over time and potentially point to new strengths and weaknesses. We will report back to the department and may publish a methods paper to share our process with other departments who may also want to do climate surveys. No identifying information will be included in any reports or publications.

***The questions we ask in this survey aim to collect your feedback on two things:*** 
1. ***Current state of affairs*** in the department/your experiences. 
2. ***Value assessment:*** Are various efforts/factors important to you, do you think the department currently values them, does the department’s level of valuing match your values, etc.? 

**Participants** 
All members of the department contribute to and experience the departmental climate. It is therefore critical that all faculty, research and administrative staff (including postdoctoral scholars), and graduate students take this survey. 

Undergraduate researchers over the age of 18 who are working in labs are encouraged to participate given their important contributions to the department and direct day-to-day involvement, but at this time, we are not soliciting feedback from Zoology & Physiology undergraduate students. We hope to expand our efforts to this population in the future.

**Survey Format**
***Survey platform:*** The survey will be administered via Qualtrics and will be split into three main sections: demographic information, a core set of questions that everyone will be asked, and a targeted set of questions specific to the respondent’s position within the department (e.g., faculty, staff, student). 
 ***Time for survey:*** We anticipate that this survey will take approximately 25 - 30 minutes. While we recognize this might seem like a lot of time, please consider that it is a comparatively small amount of time for the impact we anticipate your responses will have on the future of the department. We invite you to think of it as an opportunity to settle in with your favorite beverage and reflect on what works and what could be improved in the department.

***Survey can be taken over multiple sessions:*** Your progress can be saved as you go. The survey does not have to be completed in one sitting. Your responses will be saved and archived/submitted automatically by Qualtrics two weeks after the last time you interact with the survey. Also, there is a back button, so you can return to a section if you would like to modify or expand upon an answer in a previous section. 

**Expected Outcomes & Resulting Actions**
Survey results will likely point to departmental strengths as well as key areas in need of improvement. In both cases, we are committed to identifying and implementing actions that nurture strengths and address weaknesses in our ongoing pursuit of excellence as a department.
 ***We have the department leadership and department-wide agreement on the following:***
 1. We will celebrate what we do well and work to sustain those elements. 
2. We will be transparent about reporting and addressing issues that arise from the survey. We will take specific, concrete actions, report back regularly to faculty, staff, and students, and hold each other to a high, growth-minded standard. 
3. Department thresholds for specific actions will be established based on the information that arises from the survey results. 
4. Even a single instance of misconduct, abuse, or discrimination requires an appropriate and timely response. 
5. We anticipate issues may ebb and flow, and that some interventions may be effective while others may be well-intentioned but not prove effective. We will therefore re-evaluate our efforts and the departmental climate on a regular basis. This survey will be conducted every three years, with the expectation that our efforts will necessarily be on-going.

**Informed Consent** 
As a member of the UW Zoology and Physiology Department, you play a key role in the department’s operations and impacts. Thus, we are asking you to participate in this survey as we try to learn more about your experiences in the department. 
We will use the results of this survey to develop priorities and an action plan for enhancing the department’s operations on many fronts. We will report back to the department and publish a methods paper to share our process with other departments that may also want to do climate surveys. No identifying information will be included in any reports or publications.
Completing this survey indicates your consent as a participant in this study insofar as your responses will be analyzed. 

***Anonymity of respondents:*** Data collected is anonymous. No individual identifiers will be collected.

***Voluntary participation and optional response to individual questions:*** Participating in this study is voluntary. Your refusal to participate will involve no penalty or loss of benefits to which you are otherwise entitled, and you may discontinue participation at any time. If at any time you choose to withdraw from this study, you may do so by closing out of the browser window. Also, once you’ve started the survey you do not have to answer all of the questions. While we encourage you to answer all of the questions, you may leave questions blank at any time and continue with the survey.

***More information about the survey:*** This survey has been approved by the University of Wyoming Institutional Review Board. If you have any questions or concerns regarding this research study, feel free to contact Bethann Garramon Merkle (bmerkle@uwyo.edu) or Michael Dillon (michael.dillon@uwyo.edu).

By proceeding with this survey, you give your informed consent and verify that you are 18 years of age or older. In the actual survey, we ask you to confirm both of these before commencing the survey.

*Display This Question:*

*If What would you like to do next? = Skip to confidentiality and anonymity clarifications.*

***Responses will be anonymous to encourage candid responses.***
 However, we recognize that certain circumstances or demographic information may make respondents identifiable. Therefore, we clarify: questions may be skipped entirely and the results will only be presented in aggregate. 
 ***To ensure anonymity and your candid responses, we will take the following actions:***
1. Some demographic information (e.g., role/position in department) will be required, in order to pose you appropriate questions. All other demographic questions will be optional. 
2. Some demographics will only be reported as an aggregate of groups historically marginalized or excluded from STEM, including individuals who self-identify as nonbinary, LGBTQ+, citizens of countries other than the USA, and cultural and linguistic minorities. 
 3. Any demographics which have fewer than 10 respondents will also be reported in an aggregate fashion. 
 4. Analyses of raw data will be conducted by Gabe Barrile (recently graduated PhD student, Chalfoun and Walters Labs) and Justine Becker (postdoc, Merkle Lab). These individuals have signed a statement of confidentiality (see here [N.B. “here” was hyperlinked to a signed version available to survey participants]) which will be included in the survey report we prepare.

Future iterations of this survey will include similar practices, with modifications to further protect confidentiality as needed.

Age, Consent, and Duration

Are you over 18 years old?

- Yes
- No

Do you consent to participate in this study?

- Yes
- No

Have you been in the department more than 3 months?

*To accurately inform department planning and actions in response to this survey, we ask everyone to take the survey. Recognizing that folks who feel new to the department may have distinct perceptions, we offer this question.*

- Yes, I have been in the department 4 or more months
- No, I have been in the department under 3 full months

Sense of Belonging

This section poses questions about **sense of belonging**. We ask these questions because your experiences in this area can affect you and the department. We anticipate being able to use results from this section to inform how the department discusses and takes action on these topics.

Please indicate your level of agreement with the following statements:

|  | Strongly disagree | Disagree | Neither agree nor disagree | Agree | Strongly agree | N/A or prefer not to respond |
| --- | --- | --- | --- | --- | --- | --- |
| I feel welcome at informal social gatherings with most members of the department |  |  |  |  |  |  |
| I have opportunities to collaborate on research with other members of the department |  |  |  |  |  |  |
| The department is a place where I am able to perform up to my full potential |  |  |  |  |  |  |
| I have opportunities in the department for academic or professional success that are at least similar to those of my peers |  |  |  |  |  |  |
| My experience in the department has had a positive influence on my academic or professional growth |  |  |  |  |  |  |
| In the department, colleagues do not share important information with me that they share with others |  |  |  |  |  |  |
| I do not have access to the same mentorship opportunities as others in the department do |  |  |  |  |  |  |
| I feel others do not value my contributions to the department |  |  |  |  |  |  |
| I have considered leaving the department because I felt isolated or unwelcomed |  |  |  |  |  |  |

In the department, I feel valued and listened to by:

|  | Strongly disagree | Disagree | Neither agree nor disagree | Agree | Strongly agree | N/A or prefer not to respond |
| --- | --- | --- | --- | --- | --- | --- |
| My supervisor |  |  |  |  |  |  |
| My peers |  |  |  |  |  |  |
| My employees/students |  |  |  |  |  |  |

Select the option between each set of adjectives that best represents how you would rate the Z&P department based on **your direct experiences:**

|  | 1 | 2 | 3 | 4 | 5 |  |
| --- | --- | --- | --- | --- | --- | --- |
| Homogenous |  |  |  |  |  | Diverse |
| Racist |  |  |  |  |  | Anti-racist |
| Sexist |  |  |  |  |  | Non-sexist |
| Homophobic |  |  |  |  |  | Non-homophobic |
| Ableist |  |  |  |  |  | Non-ableist |
| Ageist |  |  |  |  |  | Non-ageist |
| Unsupportive |  |  |  |  |  | Supportive |
| Competitive |  |  |  |  |  | Cooperative |
| Disrespectful |  |  |  |  |  | Respectful |

Please indicate your level of satisfaction with the overall climate you've experienced in the last 3 years or since you've been in your current position (for new faculty/staff/students):

|  | Extremely dissatisfied | Dissatisfied | Neither dissatisfied nor satisfied | Satisfied | Extremely satisfied | N/A or prefer not to respond |
| --- | --- | --- | --- | --- | --- | --- |
| Level of satisfaction with departmental climate |  |  |  |  |  |  |

If there were two actions the department could take to improve the climate for all, what would you recommend?

________________________________________________________________

Please feel free to share any details or further thoughts you have about any of the questions/topics in this section.

________________________________________________________________

Work/Life Balance

This section poses questions about **work/life balance**.

What are the perceived expectations for the following statements:
 
*To clarify, implicit expectations might be "my supervisor works over holidays," while explicit expectations might be "my supervisor has a written policy defining the length of the work day."*

|  | No | Unsure | Implicit Expectation | Explicit Expectation | N/A or prefer not to respond |
| --- | --- | --- | --- | --- | --- |
| My supervisor expects me to work over 40 hours each week, regardless of my productivity |  |  |  |  |  |
| My peers expects me to work over 40 hours each week, regardless of my productivity |  |  |  |  |  |

Please indicate your agreement with the following statements:

|  | Strongly disagree | Disagree | Neither disagree nor agree | Agree | Strongly agree | N/A or prefer not to respond |
| --- | --- | --- | --- | --- | --- | --- |
| My supervisor expects me to take time off for official university holidays, when I am unwell, or to attend to family responsibilities |  |  |  |  |  |  |
| My peers expects me to take time off for official university holidays, when I am unwell, or to attend to family responsibilities |  |  |  |  |  |  |

Please indicate your agreement with the following statements:

|  | Strongly disagree | Disagree | Neither agree nor disagree | Agree | Strongly agree | N/A or prefer not to respond |
| --- | --- | --- | --- | --- | --- | --- |
| My supervisor clearly communicates the procedures I need to follow to take time off (e.g., vacation, personal time, sick leave, cultural/religious holidays) |  |  |  |  |  |  |
| My supervisor clearly communicates the procedures I need to follow to take an extended leave of absence (e.g., family leave or bereavement leave) |  |  |  |  |  |  |
| The department clearly communicates the procedures I need to follow to take time off (e.g., vacation, personal time, sick leave, cultural/religious holidays) |  |  |  |  |  |  |
| The department clearly communicates the procedures I need to follow to take an extended leave of absence (e.g., family leave or bereavement leave) |  |  |  |  |  |  |

Please indicate your agreement with the following statements:

|  | Strongly disagree | Disagree | Neither agree nor disagree | Agree | Strongly agree | N/A or prefer not to respond |
| --- | --- | --- | --- | --- | --- | --- |
| Personal relationships (e.g., family, a partner) are important in shaping my career decisions |  |  |  |  |  |  |
| I have been given unsolicited advice about my career and family (e.g., partner, dependents) |  |  |  |  |  |  |
| Having a dependent to care for would negatively impact my career |  |  |  |  |  |  |
| If I were to have a child, I would feel pressure to come back to work before I wanted to |  |  |  |  |  |  |

Please indicate how often each of the following has occurred in the last three years or since you've been in your current position (if less than three years):

|  | Never | Sometimes | Often | Most of the time | Almost all of the time | N/A or prefer not to respond |
| --- | --- | --- | --- | --- | --- | --- |
| How often are work-related meetings scheduled outside of standard work hours (e.g., evenings, weekends, etc.)? |  |  |  |  |  |  |
| How often do meetings outside of standard work hours negatively impact your personal life? |  |  |  |  |  |  |
| How often do conversations giving you advice focus on factors which could negatively impact your career? |  |  |  |  |  |  |

Please feel free to share any details or further thoughts you have about any of the questions/topics in this section.

________________________________________________________________

Experiences & attitudes towards JEDI

This section poses questions about **Justice, Equity, Diversity, and Inclusion (JEDI)**. We ask these questions because your experiences in this area can affect you and the department. We anticipate being able to use results from this section to inform how the department discusses and takes action on these topics.

Have you felt discriminated against in the department?

- Never
- Rarely
- Sometimes
- Often
- Very often
- N/A or prefer not to respond

*Display This Question:*

*If Have you felt discriminated against in the department? = Rarely*

*Or Have you felt discriminated against in the department? = Sometimes*

*Or Have you felt discriminated against in the department? = Often*

*Or Have you felt discriminated against in the department? = Very often*

When did this event most recently occur?

- < 1 year ago
- 1-3 years ago
- 3+ years ago
- Prefer not to answer

*Display This Question:*

*If Have you felt discriminated against in the department? = Rarely*

*Or Have you felt discriminated against in the department? = Sometimes*

*Or Have you felt discriminated against in the department? = Often*

*Or Have you felt discriminated against in the department? = Very often*

If you feel comfortable sharing, please tell us more about these experiences. For example, did you tell anyone about it and/or was any action taken?

________________________________________________________________

While at work in the department, do you ever feel expected to represent the "point of view" of your identity (e.g., your ethnicity, gender, etc.)? If you respond yes to any of these, please consider providing details at the end of this section.

|  | Never | Sometimes | Often | Most of the time | N/A or prefer not to respond |
| --- | --- | --- | --- | --- | --- |
| Academic discipline |  |  |  |  |  |
| Country of origin |  |  |  |  |  |
| Disability status |  |  |  |  |  |
| Ethnicity/cultural background |  |  |  |  |  |
| Gender identity |  |  |  |  |  |
| Religion |  |  |  |  |  |
| Sexual orientation |  |  |  |  |  |
| Socioeconomic status |  |  |  |  |  |
| Other identity |  |  |  |  |  |

*Display This Question:*

*If While at work in the department, do you ever feel expected to represent the "point of view" of yo... Other identity Is Not Empty*

*Or While at work in the department, do you ever feel expected to represent the "point of view" of yo... = Academic discipline [ Sometimes ]*

*Or While at work in the department, do you ever feel expected to represent the "point of view" of yo... = Academic discipline [ Often ]*

*Or While at work in the department, do you ever feel expected to represent the "point of view" of yo... = Academic discipline [ Most of the time ]*

*Or While at work in the department, do you ever feel expected to represent the "point of view" of yo... = Country of origin [ Sometimes ]*

*Or While at work in the department, do you ever feel expected to represent the "point of view" of yo... = Country of origin [ Often ]*

*Or While at work in the department, do you ever feel expected to represent the "point of view" of yo... = Country of origin [ Most of the time ]*

*Or While at work in the department, do you ever feel expected to represent the "point of view" of yo... = Disability status [ Sometimes ]*

*Or While at work in the department, do you ever feel expected to represent the "point of view" of yo... = Disability status [ Often ]*

*Or While at work in the department, do you ever feel expected to represent the "point of view" of yo... = Disability status [ Most of the time ]*

*Or While at work in the department, do you ever feel expected to represent the "point of view" of yo... = Ethnicity/cultural background [ Sometimes ]*

*Or While at work in the department, do you ever feel expected to represent the "point of view" of yo... = Ethnicity/cultural background [ Often ]*

*Or While at work in the department, do you ever feel expected to represent the "point of view" of yo... = Ethnicity/cultural background [ Most of the time ]*

*Or While at work in the department, do you ever feel expected to represent the "point of view" of yo... = Gender identity [ Sometimes ]*

*Or While at work in the department, do you ever feel expected to represent the "point of view" of yo... = Gender identity [ Often ]*

*Or While at work in the department, do you ever feel expected to represent the "point of view" of yo... = Gender identity [ Most of the time ]*

*Or While at work in the department, do you ever feel expected to represent the "point of view" of yo... = Religion [ Sometimes ]*

*Or While at work in the department, do you ever feel expected to represent the "point of view" of yo... = Religion [ Often ]*

*Or While at work in the department, do you ever feel expected to represent the "point of view" of yo... = Religion [ Most of the time ]*

*Or While at work in the department, do you ever feel expected to represent the "point of view" of yo... = Sexual orientation [ Sometimes ]*

*Or While at work in the department, do you ever feel expected to represent the "point of view" of yo... = Sexual orientation [ Often ]*

*Or While at work in the department, do you ever feel expected to represent the "point of view" of yo... = Sexual orientation [ Most of the time ]*

*Or While at work in the department, do you ever feel expected to represent the "point of view" of yo... = Socioeconomic status [ Sometimes ]*

*Or While at work in the department, do you ever feel expected to represent the "point of view" of yo... = Socioeconomic status [ Often ]*

*Or While at work in the department, do you ever feel expected to represent the "point of view" of yo... = Socioeconomic status [ Most of the time ]*

When did this event most recently occur? Please consider providing details at the end of this section.

- < 1 year ago
- 1-3 years ago
- 3+ years ago
- Prefer not to answer

Please indicate your level of agreement with the following statements:

|  | Strongly disagree | Disagree | Neither agree nor disagree | Agree | Strongly agree | N/A or prefer not to respond |
| --- | --- | --- | --- | --- | --- | --- |
| The department provides sufficient programs and resources to foster the success of a diverse group of students, faculty, and staff |  |  |  |  |  |  |
| The department articulates a strong commitment to JEDI |  |  |  |  |  |  |
| The department acts with a strong commitment to JEDI |  |  |  |  |  |  |
| The department should institute a committee dedicated to taking action on JEDI issues |  |  |  |  |  |  |
| The department addressing JEDI issues in STEM is important for my personal career |  |  |  |  |  |  |
| The department addressing JEDI issues in STEM is important for my field |  |  |  |  |  |  |
| Working to address JEDI issues in STEM is important for my career |  |  |  |  |  |  |
| Working to address JEDI issues in STEM is important for my field |  |  |  |  |  |  |

If there were two things the department could do to improve JEDI, what would those be and/or what would you recommend?

________________________________________________________________

Please feel free to share any details or further thoughts you have about any of the questions/topics in this section.

________________________________________________________________

Mental Health & Well-being

This section poses questions about **mental health and well-being**. We ask these questions because your experiences in this area can affect you and the department. We anticipate being able to use results from this section to inform (a) how the department discusses these topics, (b) how the department works to connect people with relevant resources, and (c) inform these services of how members of our department experience them.

Have you ever used any of the following mental health and well-being resources on-campus?

|  | Never used | Used rarely | Used Sometimes | Used often | Used very often | N/A or prefer not to respond |
| --- | --- | --- | --- | --- | --- | --- |
| University Counseling Center |  |  |  |  |  |  |
| WellSpring Counseling Center |  |  |  |  |  |  |
| Psychology Center |  |  |  |  |  |  |
| Student Health Center (non-specialist) |  |  |  |  |  |  |
| Student Health Center (mental health or psychiatric specialist) |  |  |  |  |  |  |
| Group therapy facilitated by one of the above groups |  |  |  |  |  |  |
| Other |  |  |  |  |  |  |

*Display This Question:*

*If Have you ever used any of the following mental health and well-being resources on-campus? = Never used*

*Or Have you ever used any of the following mental health and well-being resources on-campus? = N/A or prefer not to respond*

If you have **not** used one or more of these campus services, why not? Check all that apply.

- Stigma
- Financial constraints
- Wait times
- Confusions about services (i.e., I did not know I could access these services)
- Previous negative experiences with mental health/well-being services
- Previous negative experiences with UW's mental health/well-being services
- Religious or personal beliefs
- Haven't felt a need to use them
- Did not know about available services
- Other __________________________________________________

How often is mental health and well-being formally discussed at work? Please consider providing details at the end of this section.

- Never
- Rarely
- Sometimes
- Often
- Very often
- N/A or prefer not to respond

*Display This Question:*

*If How often is mental health and well-being formally discussed at work? Please consider providing d... = Rarely*

*Or How often is mental health and well-being formally discussed at work? Please consider providing d... = Sometimes*

*Or How often is mental health and well-being formally discussed at work? Please consider providing d... = Often*

*Or How often is mental health and well-being formally discussed at work? Please consider providing d... = Very often*

Who initiates these conversations? Check all that apply.

- I do
- Graduate students
- Postdocs
- Staff
- Faculty
- N/A or prefer not to respond

*Display This Question:*

*If How often is mental health and well-being formally discussed at work? Please consider providing d... = Rarely*

*Or How often is mental health and well-being formally discussed at work? Please consider providing d... = Sometimes*

*Or How often is mental health and well-being formally discussed at work? Please consider providing d... = Often*

*Or How often is mental health and well-being formally discussed at work? Please consider providing d... = Very often*

Are these conservations supported by explicit and/or implicit action?


*To clarify, implicit action might be colleagues or supervisors sharing mental health resource emails, while explicit action might be colleagues or supervisors taking actions to improve mental health & well-being of the group?*

- No
- Implicit
- Explicit
- Both
- N/A or prefer not to respond

I feel safe discussing my mental health and well-being with colleagues at work.

- Strongly disgree
- Disagree
- Neither disagree nor agree
- Agree
- Strongly agree
- N/A or prefer not to respond

Given your response above, please indicate your level of agreement with the following statements:

|  | Strongly Disagree | Disagree | Neither disagree nor agree | Agree | Strongly agree | N/A or prefer not to respond |
| --- | --- | --- | --- | --- | --- | --- |
| My mental health has been positively affected by my experiences in the Z&P department |  |  |  |  |  |  |
| My mental health has been positively affected by my experiences in the community (e.g., Laramie) |  |  |  |  |  |  |

Please feel free to share any details or further thoughts you have about any of the questions/topics in this section.

________________________________________________________________

Mental Health & Well-being

Safety

This section poses questions about **safety**. We ask these questions because your experiences in this area can affect you and the department. We anticipate being able to use results from this section to inform (a) how the department discusses and takes action on these topics and (b) how the department works to connect people with relevant campus and community resources.

In the last three years or since joining the department (if more recent), has someone ever spoken to you in a way that you feel uncomfortable in any of the following situations?

|  | Yes | No | N/A or prefer not to respond |
| --- | --- | --- | --- |
| By my advisor or someone in a position of power |  |  |  |
| When interacting with members of my research or work group |  |  |  |
| At a department event |  |  |  |
| At a conference/professional event related to my work within the Z&P department |  |  |  |
| At a social even involving members of my field |  |  |  |
| In the community |  |  |  |
| While conducting fieldwork |  |  |  |

In the last three years or since joining the department (if more recently), has someone ever crossed your physical boundaries in a way that made you feel uncomfortable in any of the following situations? 


*This could be a minor as a hand on your shoulder or a pat on the head.*

|  | Yes | No | N/A or prefer not to respond |
| --- | --- | --- | --- |
| By my advisor or someone in a position of power |  |  |  |
| When interacting with members of my research or work group |  |  |  |
| At a department event |  |  |  |
| At a conference/professional event related to my work within the Z&P department |  |  |  |
| At a social event involving members of my field |  |  |  |
| In the community |  |  |  |
| While conducting fieldwork |  |  |  |

*Display This Question:*

*If In the last three years or since joining the department (if more recent), has someone ever spoken... = Yes*

*Or In the last three years or since joining the department (if more recent), has someone ever spoken... = N/A or prefer not to respond*

*Or In the last three years or since joining the department (if more recently), has someone ever cros... = Yes*

*Or In the last three years or since joining the department (if more recently), has someone ever cros... = N/A or prefer not to respond*

If you feel comfortable sharing, please tell us more about these experiences. For example, did you tell anyone about it, did you verbally express you discomfort to that person, were any actions taken?

________________________________________________________________

Please indicate your level of agreement with the following statements:

|  | Strongly disagree | Disagree | Neither agree nor disagree | Agree | Strongly agree | N/A or prefer not to respond |
| --- | --- | --- | --- | --- | --- | --- |
| I know the resources available to me if I need to report harassment |  |  |  |  |  |  |
| I would feel comfortable reporting harassment if I experience it |  |  |  |  |  |  |
| I would feel comfortable reporting harassment if I witness it happening to someone else in the department |  |  |  |  |  |  |
| I feel confident that if I reported harassment, it would be addressed with meaningful action |  |  |  |  |  |  |

Please indicate your level of agreement with the following statements:

|  | Strongly disagree | Disagree | Neither agree nor disagree | Agree | Strongly agree | N/A or prefer not to respond |
| --- | --- | --- | --- | --- | --- | --- |
| I supervise people in the lab and/or field |  |  |  |  |  |  |
| I have formal training in how to supervise people in the lab and/or field |  |  |  |  |  |  |
| I have safety protocols in place for anyone I supervise in the lab and/or field |  |  |  |  |  |  |
| I feel comfortable implementing these safety protocols in the lab and/or the field |  |  |  |  |  |  |

Please feel free to share any details or further thoughts you have about any of the questions/topics in this section.

________________________________________________________________

Safety

Demographics - Self-Identified Demographics

This section poses questions about **demographics**, so that we are able to better understand how various demographics may experience the department. We anticipate being able to use results from this section to inform where the department needs to concentrate attention and action. For information about how we will protect your anonymity (aggregate reporting, etc.,) see the survey overview [here](https://drive.google.com/file/d/1pZsVjcsrG8jI5xCePJqVgOsF8SI7_mKO/view?usp=sharing).

How long have you been in your current position?

- Under a year
- 1-3 years
- 4-7 years
- More than 7 years

How long have you been affiliated with the Z&P department?

- Under a year
- 1-3 years
- 4-7 years
- More than 7 years

Do you identify as a member of a group that is currently or has been historically underrepresented in the STEM field?

- Yes
- No
- Unsure
- Prefer not to say

*Display This Question:*

*If Do you identify as a member of a group that is currently or has been historically underrepresente... = Yes*

*Or Do you identify as a member of a group that is currently or has been historically underrepresente... = Unsure*

If yes, please select all underrepresented identities that apply:

- Gender identity
- Sexual orientation
- Disability status
- Ethnicity/cultural heritage
- Socioeconomic status
- Yes, option not listed __________________________________________________

What's the highest level of education completed by the adult(s) who primarily raised you?

- High School/GED
- College
- Graduate School/Professional School
- Other

Do you, or did you, have family responsibilities (e.g., children, elders/parents, siblings) that you feel affect/affected your work in your current position?

- Yes
- No
- Prefer not to respond

*Display This Question:*

*If Do you, or did you, have family responsibilities (e.g., children, elders/parents, siblings) that... = Yes*

If yes, please consider elaborating on your situation.

________________________________________________________________

Demographics - Role in the Department

This section poses questions about your **role in the department**, so that you are next posed appropriate questions about your role.

Are you affiliated with any other programs on campus? Check all that apply:

- Program in Ecology
- Haub School
- WY Cooperative Research Unit
- Neuroscience Program
- Molecular and Cellular Life Sciences
- Other __________________________________________________

Which best describes your current position in the Z&P department? Only choose one.

- Faculty (including APL, APRS, Professors of Practice, and tenure-track faculty)
- Research and teaching staff (including research personnel, postdocs, etc.)
- Administrative staff (business office)
- Graduate student (current and recently graduated)
- Undergraduate student working in a research lab

*Display This Question:*

*If Which best describes your current position in the Z&P department? Only choose one. = Faculty (including APL, APRS, Professors of Practice, and tenure-track faculty)*

You've selected **Faculty (including APL, APRS, Professors of Practice, and tenure-track faculty)** as the role that best describes your current position in the Z&P department. Is that correct?

- Yes
- No, I'd like to select a different role

*Display This Question:*

*If Which best describes your current position in the Z&P department? Only choose one. = Research and teaching staff (including research personnel, postdocs, etc.)*

You've selected **Research and teaching staff (including research personnel, postdocs, etc.)** as the role that best describes your current position in the Z&P department. Is that correct?

- Yes
- No, I'd like to select a different role

*Display This Question:*

*If Which best describes your current position in the Z&P department? Only choose one. = Administrative staff (business office)*

You've selected **Administrative staff (business office)** as the role that best describes your current position in the Z&P department. Is that correct?

- Yes
- No, I'd like to select a different role

*Display This Question:*

*If Which best describes your current position in the Z&P department? Only choose one. = Graduate student (current and recently graduated)*

You've selected **Graduate student (current and recently graduated)** as the role that best describes your current position in the Z&P department. Is that correct?

- Yes
- No, I'd like to select a different role

*Display This Question:*

*If Which best describes your current position in the Z&P department? Only choose one. = Undergraduate student working in a research lab*

You've selected **Undergraduate student working in a research lab** as the role that best describes your current position in the Z&P department. Is that correct?

- Yes
- No, I'd like to select a different role

*Display This Question:*

*If You've selected Faculty (including APL, APRS, Professors of Practice, and tenure-track faculty) a... = No, I'd like to select a different role*

*Or You've selected Research and teaching staff (including research personnel, postdocs, etc.) as the... = No, I'd like to select a different role*

*Or You've selected Administrative staff (business office) as the role that best describes your curre... = No, I'd like to select a different role*

*Or You've selected Graduate student (current and recently graduated) as the role that best describes... = No, I'd like to select a different role*

*Or You've selected Undergraduate student working in a research lab as the role that best describes y... = No, I'd like to select a different role*

Which best describes your current position in the Z&P department? Only choose one.

- Faculty (including APL, APRS, Professors of Practice, and tenure-track faculty)
- Research and teaching staff (including research personnel, postdocs, etc.)
- Administrative staff (business office)
- Graduate student (current and recently graduated)
- Undergraduate student working in a research lab

Demographics - Role in the Department

Staff questions

This section poses questions about **your experience as a staff member in our department**. 


We ask these questions because your experiences in this area can affect you and the department. We anticipate being able to use results from this section to (a) inform how the department discusses and addresses these topics, (b) inform how the department works to connect people with relevant campus and community resources, and (c) inform these services of how our department members experience them.


These questions were developed based on discussions with and feedback from a committee of department staff during spring and summer 2021. 


Postdocs, for the purpose of this survey you'll be posed the staff questions. We encourage you to contribute thoughts/insights/experiences specific to mentorship and research conditions at the end of the section.

I feel what I do is valued:

|  | Strongly disagree | Disagree | Neither agree nor disagree | Agree | Strongly agree | N/A or prefer not to respond |
| --- | --- | --- | --- | --- | --- | --- |
| in the department. |  |  |  |  |  |  |
| by my supervisor. |  |  |  |  |  |  |
| by my colleagues. |  |  |  |  |  |  |
| by undergraduate students. |  |  |  |  |  |  |
| by graduate students. |  |  |  |  |  |  |
| by faculty. |  |  |  |  |  |  |

Please indicate your level of agreement with the following statements:

|  | Strongly disagree | Disagree | Neither agree nor disagree | Agree | Strongly agree | N/A or prefer not to respond |
| --- | --- | --- | --- | --- | --- | --- |
| I feel fairly compensated for my work. |  |  |  |  |  |  |
| I feel empowered to do what I am best at. |  |  |  |  |  |  |
| I feel a sense of belonging in the department broader than my immediate employment environment (e.g., lab, business office). |  |  |  |  |  |  |
| I feel I have a voice in the decisions that affect the future of the department. |  |  |  |  |  |  |

Please indicate your level of agreement with the following statements, I have room for professional development:

|  | Strongly disagree | Disagree | Neither agree nor disagree | Agree | Strongly agree | N/A or prefer not to respond |
| --- | --- | --- | --- | --- | --- | --- |
| within the department |  |  |  |  |  |  |
| within the university |  |  |  |  |  |  |

These aspects of the work environment in the department make me want to continue in my role in the department/at the university (select all that apply):

- Pay
- Opportunities for advancement
- Co-workers
- Faculty
- Graduate students
- Undergraduate students
- Department culture
- Opportunities for professional growth
- Reasonable workload
- Good work-life balance
- Flexible scheduling
- Flexibility in where I do my work
- University-provided employee benefits
- Other

Please indicate your level of agreement with the following statement on time off:

|  | Strongly disagree | Disagree | Neither agree nor disagree | Agree | Strongly agree | N/A or prefer not to respond |
| --- | --- | --- | --- | --- | --- | --- |
| I feel concerned about taking time off. |  |  |  |  |  |  |

Please indicate which of the following might be reasons why you feel concerned about taking time off:

- Work piles up too much.
- Too many emails pile up.
- I am expected to work while out of the office.
- No one has time to fill in for me.
- No one is trained to fill in for me.
- My supervisor implicitly expects me to not take time off.
- My supervisor explicitly tells me to not take time off.
- My co-workers implicitly expect me to not take time off.
- My co-workers explicitly tell me to not take time off.
- Other

Please feel free to suggest any department-level/university-level changes that could support retention. (We want to hear about even the pie-in-the-sky ideas.)

________________________________________________________________

Please feel free to share any details or further thoughts you have about any of the questions/topics in this section.

________________________________________________________________

If applicable to your position, please share any thoughts you have on research and/or mentoring conditions.

________________________________________________________________

Faculty questions

This section poses questions about **your experience as a faculty member in our department**. 


We ask these questions because your experiences in this area can affect you and the department. We anticipate being able to use results from this section to inform (a) how the department discusses and addresses these topics, (b) how the department works to connect people with relevant campus and community resources, and (c) inform these services of how our department members experience them.

Please indicate your level of agreement with the following statements regarding retention, tenure, and promotion (for non-tenure-track and tenure-track):

|  | Strongly disagree | Disagree | Neither agree nor disagree | Agree | Strongly agree | N/A or prefer not to respond |
| --- | --- | --- | --- | --- | --- | --- |
| Departmental expectations for retention, tenure, and promotion (RT&P) are clear/transparent |  |  |  |  |  |  |
| Departmental expectations for RT&P are fair |  |  |  |  |  |  |
| Departmental expectations for RT&P are on par with other institutions |  |  |  |  |  |  |
| Departmental expectations for RT&P are consistently applied |  |  |  |  |  |  |
| My evaluation of my peers for RT&P is valued by the department |  |  |  |  |  |  |
| Mentoring committees are a useful resource for pre-tenure faculty |  |  |  |  |  |  |
| Mentoring committees have specified goals, objectives, and outcomes |  |  |  |  |  |  |
| The department provides resources/support for grant writing |  |  |  |  |  |  |
| The department provides resources to support onboarding and mentoring of graduate and/or undergraduate students |  |  |  |  |  |  |
| The department provides resources/support for teaching |  |  |  |  |  |  |

Please indicate your level of agreement with the following statements regarding departmental service:

|  | Strongly disagree | Disagree | Neither agree nor disagree | Agree | Strongly agree | N/A or prefer not to respond |
| --- | --- | --- | --- | --- | --- | --- |
| Departmental service is equitable among faculty in the department |  |  |  |  |  |  |
| A few faculty do most of the service within the department |  |  |  |  |  |  |
| Service expectations of pre-tenure faculty are reasonable |  |  |  |  |  |  |

Please indicate your level of agreement with the following statements on departmental hierarchy:

|  | Strongly disagree | Disagree | Neither agree nor disagree | Agree | Strongly agree | N/A or prefer not to respond |
| --- | --- | --- | --- | --- | --- | --- |
| I feel that I must defer to more senior faculty in departmental decisions |  |  |  |  |  |  |
| I feel comfortable providing my perspective to faculty of the same rank |  |  |  |  |  |  |
| I feel comfortable providing my perspective to faculty who have been in the department for about the same time as me |  |  |  |  |  |  |
| I feel comfortable providing my perspective to faculty of a higher rank (e.g., Associate, Full) |  |  |  |  |  |  |
| I feel comfortable providing my perspective to faculty who have been in the department for longer than I have |  |  |  |  |  |  |
| I interact regularly with faculty of the same rank |  |  |  |  |  |  |
| I interact regularly with faculty of a higher rank (e.g., Associate, Full) |  |  |  |  |  |  |
| I interact regularly with individuals in the department who are not faculty (e.g., undergraduate students, graduate students, staff) |  |  |  |  |  |  |

Please indicate your level of agreement with the following statements on collaboration:

|  | Strongly disagree | Disagree | Neither agree nor disagree | Agree | Strongly agree | N/A or prefer not to respond |
| --- | --- | --- | --- | --- | --- | --- |
| Collaboration among faculty in the department is encouraged |  |  |  |  |  |  |
| I can identify one or more colleagues in the department with whom I have collaborated or could collaborate, leading to a product (e.g., research, course development) |  |  |  |  |  |  |
| I feel comfortable recommending departmental colleagues to my students (e.g., mentoring, committees, collaborations, etc.) |  |  |  |  |  |  |

Please feel free to share any details or further thoughts you have about any of the questions/topics in this section.

________________________________________________________________

Any other thoughts/comments about the general climate or being a faculty member in the Z&P Department?

________________________________________________________________

Graduate student questions

This section poses questions about **your experience as a graduate student in our department**. 


We ask these questions because your experiences in this area can affect you and the department. We anticipate being able to use results from this section to inform (a) how the department discusses and addresses these topics, (b) how the department works to connect people with relevant campus and community resources, and (c) inform these services of how our department members experience them.

My graduate advisor encourages me to:

|  | Strongly disagree | Disagree | Neither agree nor disagree | Agree | Strongly agree | N/A or prefer not to respond |
| --- | --- | --- | --- | --- | --- | --- |
| Attend professional conferences |  |  |  |  |  |  |
| Present research at professional conferences |  |  |  |  |  |  |
| Co-author in refereed journals |  |  |  |  |  |  |
| Publish as first author in a refereed journal |  |  |  |  |  |  |
| Serve on an official committee (Z&P, PiE, etc.) |  |  |  |  |  |  |
| Participate in outreach opportunities |  |  |  |  |  |  |
| Participate in JEDI activities |  |  |  |  |  |  |
| Mentor undergraduates |  |  |  |  |  |  |
| Develop strong relationships with other academic/professional mentors (e.g., committee members, postdocs, mentors outside of department or university) |  |  |  |  |  |  |

My graduate advisor:

|  | Strongly disagree | Disagree | Neither agree nor disagree | Agree | Strongly agree | N/A or prefer not to respond |
| --- | --- | --- | --- | --- | --- | --- |
| Is knowledgeable about UW and Z&P formal degree requirements |  |  |  |  |  |  |
| Serves as my advocate when necessary |  |  |  |  |  |  |
| Helps me secure financial support for my graduate work |  |  |  |  |  |  |
| Gives me constructive feedback on my work |  |  |  |  |  |  |
| Returns my work promptly (within 1-2 weeks) |  |  |  |  |  |  |
| Promotes my professional development |  |  |  |  |  |  |
| Provides helpful advice about career paths within academia |  |  |  |  |  |  |
| Provides helpful advice about career paths outside academia |  |  |  |  |  |  |
| Assists me in my search for employment |  |  |  |  |  |  |
| Guides me through major academic milestones (e.g., proposal, comprehensive exams, defense, etc.) |  |  |  |  |  |  |
| Has created a supportive and productive lab culture |  |  |  |  |  |  |
| Overall, performs their mentoring/advising roll well |  |  |  |  |  |  |

Please indicate your level of agreement with the following statements:

|  | Strongly disagree | Disagree | Neither agree nor disagree | Agree | Strongly agree | N/A or prefer not to respond |
| --- | --- | --- | --- | --- | --- | --- |
| I received accurate information about my funding, stipend, and benefits when hired |  |  |  |  |  |  |
| My advisor provides additional funding when needed (e.g., summer salary, conference travel) |  |  |  |  |  |  |
| My advisor helps me apply for my own funding via grants or scholarships |  |  |  |  |  |  |
| My graduate stipend is sufficient to cover my living expenses (e.g., rent, food, medical expenses) |  |  |  |  |  |  |
| I was issued a personal university P-Card |  |  |  |  |  |  |
| My advisor expects me to pay out-of-pocket for costs related to my research upfront and be reimbursed later |  |  |  |  |  |  |
| I have extra financial obligations (e.g., supporting family members/dependents, paying off debts, etc.) |  |  |  |  |  |  |
| The compensation I receive is fair for the work I do |  |  |  |  |  |  |
| The compensation I receive is on par with what I would expect to receive in a comparable program elsewhere |  |  |  |  |  |  |

Please feel free to share any details or further thoughts you have about any of the questions/topics in this section.

________________________________________________________________

Any other thoughts/comments about the general climate or being a graduate student in the Z&P Department?

________________________________________________________________
