# Supplementary material for: Equity, community, and accountability: Leveraging a department-level climate survey as a tool for action
Source: PLoS One. 2023 Aug 17;18(8):e0290065. doi: 10.1371/journal.pone.0290065 (PMC10434968; doi:10.1371/journal.pone.0290065)
Supplement: S2 File — (DOCX) [file pone.0290065.s002.docx]

**Supplement 2**

**Journal Name:** *PLOS ONE*

**Manuscript Title**: Equity, community, and accountability: leveraging a department-level climate survey as a tool for action

**List of Authors:**

Gabriel M. Barrile, Riley F. Bernard, Rebecca C. Wilcox, Justine A. Becker, Michael E. Dillon, Rebecca R. Thomas-Kuzilik, Sara P. Bombaci, and Bethann Garramon Merkle

**Corresponding Author Email:** [gbarrile15@gmail.com](mailto:gbarrile15@gmail.com)

**Supplement 2 – Inception of climate survey and related efforts**

Our department-level climate efforts started nearly a year before the development of our survey. We present them here as the foundations of these efforts substantially informed our survey work, and indeed, instigated it (summarized in Figure 1 on the next page). We also recount the origins of this effort in recognition that some of our initial activities may be more viable for departments that are interested in climate work but not yet ready to invest in a departmental climate survey.

**
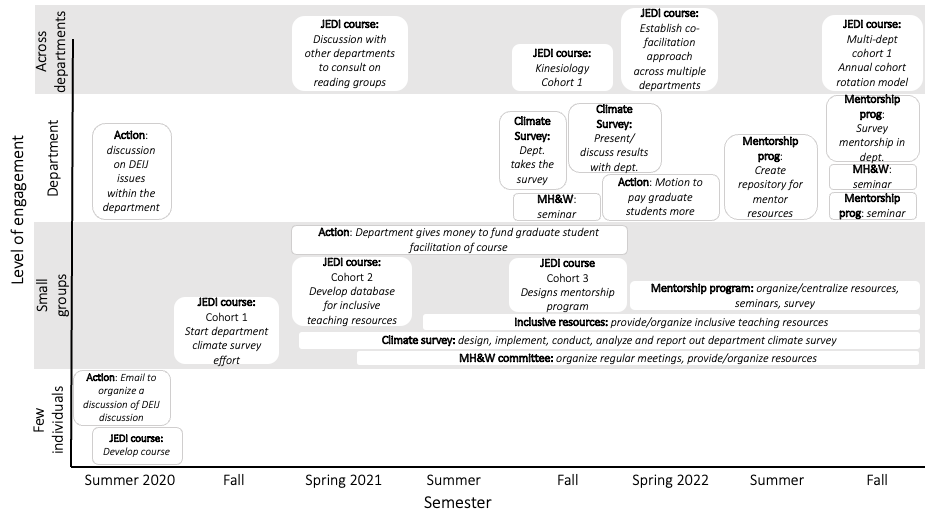
**

**Figure 1.** Timeline of actions and efforts stemming from initial diversity, equity, inclusion, and justice (DEIJ) discussions and their level of engagement. Each of the initial three DEIJ course cohorts developed an action item to help address DEIJ issues in the department. Cohort one created the climate survey (*climate survey*) outlined in this manuscript. Cohort two developed a database for inclusive teaching resources (*inclusive resources*), and cohort three established a collection of mentorship resources (*mentorship program*) for faculty and graduate students. This timeline also includes the inception of and subsequent actions of the departmental Mental Health and Wellness Group (MH&W). This group formed independently of the reading groups, but is highly relevant to improving workplace climate in the department.

Our department had neither an active agenda nor a statement addressing diversity, equity, inclusion, and justice (hereafter, DEIJ) matters prior to summer 2020. After the national justice movements during the summer of 2020, there were quiet side conversations, but no action was initially proposed or taken by the department. However, one faculty member (Merkle) issued an email invitation to faculty, graduate students, and staff to join a facilitated, virtual discussion on the day of the nationwide academic walkout (10 June 2020; https://www.shutdownstem.com/) in support of Black Lives Matter. The discussion and subsequent meetings followed a carefully developed agenda and discussion process (see *Facilitation plan* section below) which held space for people to voice concerns, identify issues warranting attention within the scope of influence of the department, and work collectively toward possible next steps. The facilitation process also recognized and accounted for the reality that personnel in the department vary widely in their comfort, awareness, and education on social justice matters. We emerged with a list of priorities and commitments from individuals to take specific actions, some of which led to the creation of various groups and actions within the department (Figure 1).

One of the actions was our starting point for this survey: a reading group on DEIJ principles in science, technology, engineering, and math (STEM) developed and led by co-authors Merkle and Dillon in fall 2020. At the time, we were using the acronym JEDI; we have since shifted to DEIJ for our broader work, but the reading group retains the name JEDI for recognition and consistency in the department. We issued the invitation via email (see Box 1 in the *Reading group* section, below).

We developed this reading group structure with two goals in mind, one around process and one around intent. Our process goal was to build capacity, starting with shared vocabulary. As for intent, our mutual agreement within the reading group was to read and learn toward implementing change, and to hold ourselves accountable to more than reading. We set this goal in recognition that many within our field are consistently dealing with elements of systemic abelism, homophobia, racism, sexism, and socio-economic disparity; in that context, reading is an important step but is not enough. A semester agenda and complete reading list are provided in the *Reading group* section below. By semester’s end, the group decided to pursue a collective action project. From several possibilities, we decided to focus on the development of a climate survey for the department, for the reasons outlined in the Introduction of the main manuscript. Our survey goals were, like many surveys, both descriptive and explanatory.

*Facilitation plan*

Here is a summary of our facilitation plan for initial discussions around anti-racism and DEIJ efforts in our department:

**1. Initial email (ours was sent by co-author Merkle) on 9 June 2020. In addition to some discussion of a bystander/anti-harassment training opportunity, the email offered the following invitation.**

If you want to be part of on-going conversations about what else we can do, as a department, we could potentially have a first conversation/meeting about this tomorrow morning. I am planning to observe the #ShutdownSTEM #Strike4BlackLives tomorrow. This day of action is meant to a) de-prioritize general academic work in recognition of so many people who are struggling to cope with their lives being at risk, and b) instead use the day tomorrow to learn and make action plans for individual and institutional actions and accountability toward anti-racist growth. To that end, I propose a first discussion could take place tomorrow morning, and we could move forward from there. If you are interested in being part of such a conversation, please let me know. I'll send out a Zoom link for a conversation at 10:00 AM tomorrow (Wednesday, 10 June) and a couple of suggested readings, to give us a place to start from. If you are interested, but unable to join tomorrow, just let me know.

**2. Follow-up email (from co-author Merkle) with primer readings and agenda for 10 June 2020 meeting**

Hello all -

I'm bumping this up in your inboxes, in case you missed it yesterday. In order to keep moving on this work, I proposed that those who are available could meet this morning, to at least discuss what initiating a department effort could look like.

To that end:

1. Here is a Zoom link for a call at 10:00 am today (call-in details are below). If you are interested but unable to join today, please let me know. I'll keep posting about this to the whole department, so that folks stay in the loop.
2. Likely Agenda - please let me know if you'd like to see something added.

- Zoom link is for 10:00-11:30 AM; we can use as much of that time as we need/people are available.
- Why we are here (in chat)
- What we hope to accomplish (in chat)
- Ideas for what the department can do
- What we can individually commit to in the next month, next semester, longer-term (in chat)
- Anything else?
- I plan to capture the discussion and chat (anonymously) and create a shared document that we can keep working from. If you have things to contribute to it but won't be able to join us this morning, feel free to let me know!

1. Here are a couple of things to read to get us started on a common footing. If you can read any of them before we meet, that will be awesome, but obviously, this is a short turn-around.

- <https://500womenscientists.org/updates/2020/6/1/take-action>
- [Why You Need to Stop Saying "All Lives Matter"](https://www.harpersbazaar.com/culture/politics/a27075028/black-lives-matter-explained/)
- [Template for letter to employer re anti-racism accountability](https://wyocoopunit.box.com/s/8w7uzgn0x478gxsiips26vl4od4qy59m)

Looking forward to building and maintaining this work, together, in our department!

Bethann

P.S. In solidarity with the Black Lives Matter movement, I am observing today's #ShutDownSTEM #Strike4BlackLives, and only working on related things today. If your message is not about anti-racism or efforts to make science and academia more inclusive and genuinely safe, I will be responding to regular emails tomorrow. Please join the effort, if you are able. Details: <https://www.shutdownstem.com/>

**3. Facilitation notes from initial meeting/discussion:**

Context for departments considering such a meeting: Two major issues may arise in early (and on-going) work on DEIJ topics: (a) time constraints and (b) unevenness of people’s previous training and sensitivity to DEIJ issues. The initial conversations were facilitated as described below in order to attend to both of these issues in a fashion that created space for people to voice their perspectives while not (a) letting anyone voice dominate or (b) well-intentioned or malicious actors to perpetrate macro- or micro-aggressions. In essence, rather than opening the floor (on zoom) to open discussion, all participants responded to prompts in either the chat or an anonymous Google Doc.

Prompt 1: Why we are here? Please enter into the chat why you are participating in this conversation today.

- Open-ended to allow for wherever people are at that moment.
- Used chat to encourages accountability through identifiable statements.

Statements from the chat were then copied over to a notes doc (anonymized), and a word cloud was created after the meeting and provided to the department listservs in a follow-up/next steps email.


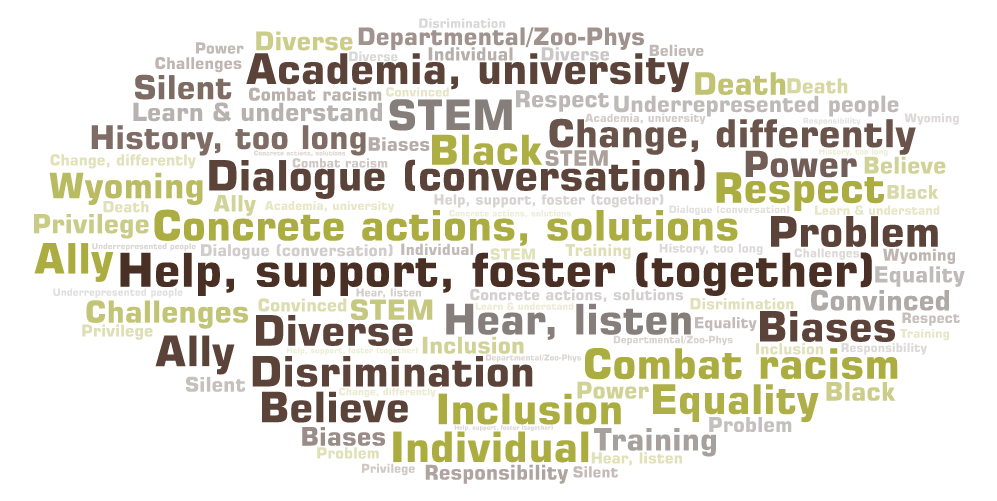


Prompt 2: What we hope to accomplish. Using the Zoom chat, please state what you would hope to accomplish through work in the department on DEIJ issues.

- Open-ended to allow for wherever people are at that moment.
- Used chat to encourages accountability through identifiable statements.

Statements from the chat were then copied over to a notes doc (anonymized), and a word cloud was created after the meeting and provided to the department listservs in a follow-up/next steps email.


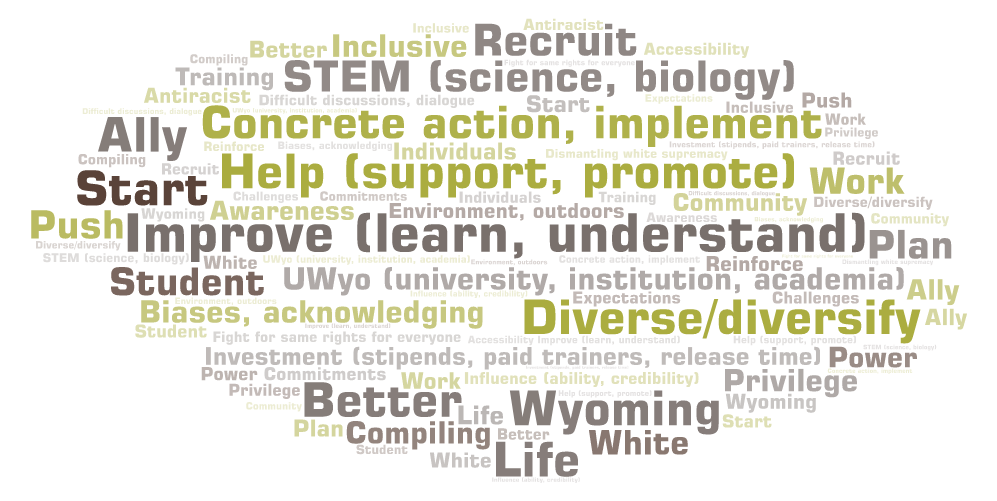


Prompt 3: Considering our motivations for being here, and what we hope to change, let’s discuss concrete, actionable ideas for what the department can do.

- This was facilitated as an open discussion.
- The facilitator (co-author Merkle) reserved the right/option to direct people into breakout rooms or anonymous Google Doc if someone started to dominate the discussion. In our case, that was not necessary, but it is a valuable tool for facilitators nonetheless. An analog option would be to ask people to write notes on notecards and/or discuss in small groups before reporting out to the group.

Our discussion on this topic resulted in a 3-page list of possible actions. They fit loosely into the following categories.

| **Category** | **Examples Suggested by Participants** | **Concrete Actions Selected by Participants** |
| --- | --- | --- |
| Trainings | 1. Green Dot, 2. Bystander 3. DEIJ trainings from experts on and off campus 4. Working trainings into department seminar series, etc. | 1. Schedule (Merkle) and attend (department) Green Dot training for the department’s faculty, staff, postdocs, and grad students   Outcomes: The Green Dot training was scheduled and well-attended. No follow-up has been conducted. Several students and faculty, primarily from one research group, volunteered to develop resources and a lab meeting theme around these topics. They did so and shared materials with the rest of the department. No follow-up has been conducted. |
| Diversification | 1. Diversify invited speaker lists for invited talks and seminars 2. Diversify content in courses 3. Look for department-level (and beyond) funds to support faculty efforts to diversify courses/syllabi and support invited speakers at class level | 1. Identify 1-2 people we would want to invite 2. Emphasize speakers who focus on DEIJ issues. 3. Boost early career folks by prioritizing them for seminar invitations. 4. Push for department to approve honoraria (in lieu of travel expenses, since seminar is virtual during pandemic). 5. Share with department the speaker databases designed to support these efforts.   Outcomes: One faculty member and one student signed up; however, these actions were not implemented at the time. Later, in spring 2021, the second JEDI reading group cohort developed a set of resources specifically targeted at this work and shared them with the whole department. No follow-up has been conducted. |
| Recruitment - making department, UW, and Laramie a safer and more welcoming place | 1. REU funding/programs 2. Scholarships to support diverse recruiting 3. Outreach toward diverse demographics in the state 4. Recruit in cohorts & run cohort-level programs and courses 5. Offer DEIJ and anti-racism courses | 1. Establish a mechanism for telling current department undergrads about research opportunities in the department   Outcomes: One faculty member volunteered to develop a resource document but did not take action. Two faculty volunteered to develop and host a reading group/course for the department. That was initiated by co-authors Merkle and Dillon in fall 2020 and is reported on in more detail elsewhere in this manuscript. Somewhat independent of the initial conversation, three faculty initiated a cohort course for incoming master’s students in fall 2022. (Such a course already existed for ecology PhD students in the department.) Several faculty members and one student were involved in two separate REU funding proposals, both of which were unsuccessful. In 2021-2022, a group of faculty and students developed a set of mentoring resources which were shared with the department. No follow-up has been conducted. In 2021, a group of faculty and students convened an *ad hoc* committee which was eventually approved as a standing committee focused on mental health and wellness. Their activities have included inviting a relevant speaker for each semester’s seminar series and establishing a food pantry to support students and staff experiencing food insecurity. No formal follow-up has been conducted on these activities. |
| Learn from others’ experience and insights | 1. Efforts must be informed by BIPOC insights and wisdom. But don’t overburden them with our learning process. 2. Others on campus have been doing this work - we can learn from them. 3. What is the university doing about this?    1. Reach out to UW’s DEI office and director.    2. Missed opportunity - department’s required 7-year review (2019). Panel was all-White and not apparently diverse in other ways; limited discussion of diversity.    3. How can we institutionalize scrutinizing this in our department?    4. Consider a department-level climate survey like the UW survey. 4. Build department’s capacity toward DEIJ 5. Be aware of and responsive to intersectional issues and career stage issues. | 1. No initial actions/commitments were identified.   Outcomes: as reported extensively in this manuscript, the fall 2020 JEDI reading group (all co-authors) initiated a climate survey process. |
| How to identify issues in our department? | 1. Surveys? 2. Train peer facilitators to run focus groups? 3. Survey external folks about department? | 1. No initial actions/commitments were identified.   Outcomes: as reported extensively in this manuscript, the fall 2020 JEDI reading group (all co-authors) initiated a climate survey process. |
| Supporting individuals/reinforce department expectations and values on a daily basis | 1. How to shut down racist/sexist jokes? 2. Trainings for all career levels, then support for implementing in classrooms 3. Role-play training? 4. Some labs aren’t very safe. How to work with them (especially PIs)? | 1. No initial actions were identified.   Outcomes: as reported extensively in this manuscript, the fall 2020 JEDI reading group (all co-authors) initiated a climate survey process. |

Prompt 4: What we can individually commit to in the next month, next semester, longer-term?

- At the close of the meeting, participants were asked to specifically identify an action from the list of ideas that they were willing to work on. Those commitments and brief reports of outcomes are detailed in column 3 of the table.

**4. Second meeting - two weeks after initial meeting**

A follow-up email summarizing the initial meeting was sent afterward, to help connect people who could not attend the first meeting. A second meeting was also scheduled and announced in that email.

Agenda items for the second meeting included:

- Touching base on initial ideas
- Discussing new ideas ones:
  - Establishing an ESA SEEDs chapter at UW - there was considerable interest in this idea, led by two graduate students who are SEEDs alumni. Ultimately, however, the students were unable to prioritize getting this going, and it has not been pursued.
  - Connecting undergrads with department values/commitment to anti-racism - there was extensive discussion of this and how to accomplish it. One faculty member volunteered to take the lead on developing regular information sessions for undergrads about research opportunities in the department. Several faculty and grad students volunteered to support. However, after one follow-up meeting, no action has been taken to date.
- Making a plan for progress on those
- Big-picture planning/ideas

*Reading group*

Below is a summary of our JEDI reading group logistics and the complete reading list.

| **Box 1.** Email invitation for initial JEDI reading group |
| --- |
| We hope for a mix of faculty, staff, and graduate students (1 seminar credit is optional). In order to build a cohort of folks with shared language and concrete, actionable plans to move our department forward, we ask folks to commit to attending every session or to wait for a future semester.  Guiding Spirit  “we need a r/evolution of the mind. we need a r/evolution of the heart. we need a r/evolution of the spirit… we need to be weapons of mass construction. weapons of mass love. it’s not enough just to change the system. we need to change ourselves.” — Assata Shakuur  Schedule   - Friday, August 28, 11:30 am-1:00 pm; initial workshop on Collaborative Conversation. - Bi-weekly discussions, Thursdays, 12:00-1:00 pm, starting September 8. - The focus will be on (a) reading toward action (collective and individual) and (b) digestible materials. We will emphasize articles, podcasts, and films (rather than books). As for action - the idea is to work beyond learning and talking, toward changes at whatever level we can impact. - After the first 5 sessions, participants will assume facilitation responsibilities. Part of our work in the first 2 weeks will be to identify a facilitation partner, sign up for a week, and start working ahead to identify materials the group will work through in prep for your week’s discussion. |

The reading group agenda was as follows:

Week 1: Collaborative Communication workshop led by UW experts in this dialogue process

Week 2: Collective Goal-Setting

Participants were encouraged to review at least 2 of the items listed below in preparation for this week’s discussion.

- Watch:
  - Act.TV’s “Systemic Racism Explained” video. ([link](https://youtu.be/YrHIQIO_bdQ); 4.5 minutes)
  - Andrade, M. 2018. Challenges around Unconscious Bias. ([link](https://youtu.be/0jkA7_yyUzw); 4.5 minutes)
- Read:
  - Johnson, T. 2020. When Black People Are In Pain, White People Just Join Book Clubs. *Washington Post*. ([link](https://drive.google.com/file/d/1SYgQoJGDxds-H9UfBfiRWG_UW5SWW98K/view?usp=sharing))
  - McWhorter, J. 2020. Academics are Really, Really Worried about their Freedom. *The Atlantic*. ([link](https://drive.google.com/file/d/1w1sHTYKFJedN-8VpJoFXvzuOioh2sza_/view?usp=sharing))
  - Johnson, M.Z. 2015. 3 Things To Consider When Choosing Between Calling Someone Out Or Calling Them In. *Everyday Feminism*. ([link](https://drive.google.com/file/d/1sqPp9lrXjuwl-eK2TM_k69NXe_-uVhoT/view?usp=sharing))

Week 3: Systemic Racism

The guiding principle of this week was summarized in this cartoon, provided to all participants along with the reading list.

- Read:
  - Racism (pick one of these):
    - SURJ. 2020. Racism 101: Understanding Race and Racism. Showing Up for Racial Justice. ([link](https://www.showingupforracialjustice.org/racism-101.html)). *Be sure to click the dropdowns for interpersonal, institutional, and structural racism.*
    - Martinez, E.B. What is White Supremacy? Catalyst Project. [www.collectiveliberation.org](http://www.collectiveliberation.org). ([link to PDF](https://drive.google.com/file/d/1D1lyw4O1yAAmuWN2sCFXtWvJI7RG3vR4/view?usp=sharing))
  - Privilege (pick two of these)
    - Citizenship Privilege & Immigrant Ally Basics. Coloradans for Immigrant Rights, American Friends Service Committee. ([link to PDF](https://drive.google.com/file/d/1enwPsXiQKT3nAy9J8BLwmwHdA6qPEkIr/view?usp=sharing))
    - Peggy McIntosh - classic article on white privilege ([link](https://www.racialequitytools.org/resourcefiles/mcintosh.pdf))
    - SURJ. 2020. White Privilege & Benefits ([link](https://www.showingupforracialjustice.org/white-privilege.html))
    - Odekunle 2020, Dismantling systemic racism in science ([link](https://science.sciencemag.org/content/369/6505/780.3?intcmp=trendmd-sci))
- Optional - listen (59 min.):
  - [http://opinionsciencepodcast.com/episode/systemic-racism-phia-salter/](http://opinionsciencepodcast.com/episode/systemic-racism-phia-salter/?fbclid=IwAR3-UCxLkr0hHCIoTqBIY8QoIPFhgThSQP9RNpTq4c28TMBjKQn7cSK-4eY)

Week 4: Microaggression

Participants were encouraged to review at least 2 of the items listed below in preparation for this week’s discussion.

- Listen (with transcript):
  - <https://www.npr.org/2020/06/08/872371063/microaggressions-are-a-big-deal-how-to-talk-them-out-and-when-to-walk-away>
- Watch:
  - Harris III, F., and J.L. Wood. 2020. Responding Racial Bias and Microaggressions in Online Environments. Cora Learning. ([YouTube link](https://youtu.be/9cEWQJ32nqU); 1 hour)
- Read:
  - Ganote et al_2016_Responding to Microaggressions with Microresistance_a Framework for Consideration ([link](https://drive.google.com/file/d/1kGPr8_i20LBB-GQRM2NCOAjg6yPBOW7y/view?usp=sharing))
  - Cadet, D. 2020. Your Black colleagues may look like they’re okay - chances are they’re not. *Refinery 29.* ([link](https://drive.google.com/file/d/1i2lZumm8HJkcIXjTKjUYyXj2wd4obkJ4/view?usp=sharing))
  - Roberts, J. 2020. White Academia: Do Better. 10 Things White Academics Can Do. *Medium: The Faculty Lounge*. ([link](https://drive.google.com/file/d/1Dx8JDySL6T8jcIdnuTapN9uxJyifPn31/view?usp=sharing))

Week 5: Decolonizing STEM. We ultimately did not shift to participant-facilitated discussions; rather, we identified two additional topics, then shifted to a full-group focus on the action project the group selected from several ideas.

Participants were encouraged to review at least 2 of the items listed below in preparation for this week’s discussion.

- Listen:
  - <https://soundcloud.com/phdivas/can-we-be-academics-and-activists>
- Read:
  - Diep, F. 2020. How Higher Ed Can Fight Racism: ‘Speak Up When It’s Hard’. *Chronicle of Higher Education*. ([link](https://drive.google.com/file/d/1zIOhDBB0jxxzX1Zic8JDYQRsnRyrv5XC/view?usp=sharing))
  - O’Brien, L.T., H.L. Bart & D.M. Garcia. 2020. Why are there so few ethnic minorities in ecology and evolutionary biology? Challenges to inclusion and the role of sense of belonging. *Social Psychology of Education* 23: 449–477. ([link](https://link.springer.com/article/10.1007%2Fs11218-019-09538-x))
  - Apkarian, N., K. Quardokus Fisher, and B.A. Burt. 2019. Inclusive Approaches to Reviewing Scholarship: A New Guide. Accelerating Systemic Change in STEM Higher Education. ([link](https://drive.google.com/file/d/17CeZqK4yqt1JA9V_78bWhqGvLE-KFxGB/view?usp=sharing))
  - Schell et al., Recreating Wakanda, Nature Ecology and Evolution ([link](https://www.nature.com/articles/s41559-020-1266-7))
  - Report on this, long, but maybe helpful: <https://www.aplu.org/library/strengthening-pathways-to-faculty-careers-in-stem-recommendations-for-systemic-change-to-support-underrepresented-groups/File>
  - Early Career Reviewer Database: <https://sites.google.com/view/ecrdatabase/add-yourself?authuser=0>
  - [Review of book on Equity in Science](https://www.nature.com/articles/d41586-020-02940-y?WT.ec_id=NATURE-20201022&utm_source=nature_etoc&utm_medium=email&utm_campaign=20201022&sap-outbound-id=9C60342BC6C75F252B2C413B6377FB114CDFCC62)

Week 6: Antiracist Pedagogical Approaches

Participants received the following prompt: Be thinking about which project idea you might want to work on during the last three weeks of our semester together: seminar course planning (and associated credit requirements, co-teaching credit, etc.) or climate survey prep. We might be able to divide and conquer, too.

Participants were encouraged to review at least 2 of the items listed below in preparation for this week’s discussion.

- Listen:
  - Young, R. 2020. How Power Erodes Empathy, and the Steps We Can Take to Rebuild It. ([link](https://www.wbur.org/hereandnow/2020/07/09/jamil-zaki-empathy-power); 11 minutes)
- Watch:
- Read:
  - [Project Biodiversify](https://projectbiodiversify.org/) - Tools to diversify and humanize biology; a repository of teaching materials and methods aimed at enhancing human diversity and inclusivity in biology courses.
  - Artze-Vega, I. 2020. Channel Outrage and Disillusionment Into Action. *Inside Higher Education*. ([link](https://drive.google.com/file/d/1Fx8CKYrPkfm6_jTB7OEUm7Zs3mBPMnTE/view?usp=sharing))
  - Brazas, C. and C. McGeehan. 2020. What White Colleagues Need to Understand. *Teaching Tolerance* 64. ([link](https://drive.google.com/file/d/1aXW-E1ysFuT69hwwCruNDbDRWNmy8Fly/view?usp=sharing))
  - Pitts, J. 2020. Teaching as Activism, Teaching as Care. *Teaching Tolerance* 64. ([link](https://drive.google.com/file/d/1TVVs6NAfQMl7zKInuus-IIeEuXiLLpi_/view?usp=sharing))
  - McMurtrie, B. 2020. What does Trauma-Informed Teaching Look Like? *Chronicle of Higher Education*. ([link](https://drive.google.com/file/d/12adDuAvR54AZjfVL7ADscIP8KRfwlV1t/view?usp=sharing))

Week 7: Planning for next reading group and next steps for deciding on a project

Week 8: Establishing work plan and scope of group project (department climate survey)
