# Supplementary material for: Equity, community, and accountability: Leveraging a department-level climate survey as a tool for action
Source: PLoS One. 2023 Aug 17;18(8):e0290065. doi: 10.1371/journal.pone.0290065 (PMC10434968; doi:10.1371/journal.pone.0290065)
Supplement: S3 File — (DOCX) [file pone.0290065.s003.docx]

**Supplement 3**

**Journal Name:** *PLOS ONE*

**Manuscript Title**: Equity, community, and accountability: leveraging a department-level climate survey as a tool for action

**List of Authors:**

Gabriel M. Barrile, Riley F. Bernard, Rebecca C. Wilcox, Justine A. Becker, Michael E. Dillon, Rebecca R. Thomas-Kuzilik, Sara P. Bombaci, and Bethann Garramon Merkle

**Corresponding Author Email:** [gbarrile15@gmail.com](mailto:gbarrile15@gmail.com)

**Supplement 3 – 2021 ZP Climate Survey: Context, Consent, Definitions & Acronyms**

We have provided this document as a duplicate reference for you as you take the survey. This document will also be available for your reference after the survey.

If you have not taken the survey, we are aiming for a 100% response rate to accurately inform department planning and actions.

**Document Table of Contents**

[Introduction](#_gwfdy4kq90vj) 1

[Context](#_dkwduxwbzkbs) 2

[Goals](#_x9t8jc68oz1e) 2

[Participants](#_mxo5calwjpe2) 2

[Survey Format](#_ikh9e2yd3b1t) 2

[Expected Outcomes & Resulting Actions](#_w3k24snxrk7r) 2

[Confidentiality and Anonymity](#_sk64lfs8og8l) 3

[Informed Consent](#_dz35xwrj1lsa) 4

[Definitions & Acronyms](#_jet91g13mcbr) 4

## Introduction

**We think this context is important for you as you work through the survey.**

***The overarching goal of this survey*** is to characterize departmental climate (e.g., the way that people experience being part of the department).

Climate is a key determinant of diverse aspects of success in work settings, including in academia. It will be valuable for us all to understand this experience from the perspectives of students, staff, and faculty.

By identifying areas of excellence as well as areas in need of improvement we can target specific future actions to build on our successes and tackle any issues that emerge.

Regular administration of the survey provides a useful tool for tracking department climate over time and for measuring the effectiveness of actions we take to improve the department. Therefore, we plan to administer this survey on a 3-year rotation.

## Bonuses for taking survey

The Z&P Department is sponsoring 10 Downtown Laramie gift certificates ($20 each), with at least 5 reserved for graduate students. After you complete the survey, you will be prompted to submit your name and email address via a separate Google form (to preserve anonymity in survey submissions) to be entered into a drawing for one of the gift cards!

## Context

This is an ideal time to implement this survey, given: (a) the department was externally reviewed in 2020, (b) our programs are currently under review as part of university-wide strategic planning, and (c) numerous recent efforts are underway by faculty, staff, and students to enhance our department.

These efforts include strengthening the graduate program with a new charge for the Graduate Advisory Board and standardized processes, bolstering undergraduate research, jumpstarting fundraising, ongoing action-oriented justice, equity, diversity, and inclusion (JEDI) reading groups, and interest in enhancing community between disciplines and career levels.

If anything, the current reorganization discussion makes this all the more pressing. We want to identify key aspects of the departmental climate we value and take those positive aspects of ZP into the future. We also intend to use “need to change” results to inform the future of ZP.

## Goals

This climate survey will inform all of these efforts and, critically, will be an ongoing process with results driving action. Subsequent surveys (every 3 years) will evaluate the effectiveness of actions, track climate over time and potentially point to new strengths and weaknesses. We will report back to the department and may publish a methods paper to share our process with other departments who may also want to do climate surveys. No identifying information will be included in any reports or publications.

***The questions we ask in this survey aim to collect your feedback on two things:***

- ***Current state of affairs*** in the department/your experiences.
- ***Value assessment:*** Are various efforts/factors important to you, do you think the department currently values them, does the department’s level of valuing match your values, etc.?

## Participants

All members of the department contribute to and experience the departmental climate. It is therefore critical that all faculty, research and administrative staff (including postdoctoral scholars), and graduate students take this survey.

Undergraduate researchers over the age of 18 who are working in labs are encouraged to participate given their important contributions to the department and direct day-to-day involvement, but at this time, we are not soliciting feedback from Zoology & Physiology undergraduate students. We hope to expand our efforts to this population in the future.

## Survey Format

*Survey platform:* The survey will be administered via Qualtrics and will be split into three main sections: demographic information, a core set of questions that everyone will be asked, and a targeted set of questions specific to the respondent’s position within the department (e.g., faculty, staff, student).

*Time for survey:* We anticipate that this survey will take approximately 25 - 30 minutes. While we recognize this might seem like a lot of time, please consider that it is a comparatively small amount of time for the impact we anticipate your responses will have on the future of the department. We invite you to think of it as an opportunity to settle in with your favorite beverage and reflect on what works and what could be improved in the department.

*Survey can be taken over multiple sessions:* Your progress can be saved as you go. The survey does not have to be completed in one sitting. Your responses will be saved and archived/submitted automatically by Qualtrics two weeks after the last time you interact with the survey. Also, there is a back button, so you can return to a section if you would like to modify or expand upon an answer in a previous section.

## Expected Outcomes & Resulting Actions

Survey results will likely point to departmental strengths as well as key areas in need of improvement. In both cases, we are committed to identifying and implementing actions that nurture strengths and address weaknesses in our ongoing pursuit of excellence as a department.

We have the department leadership and department-wide agreement on the following:

- We will celebrate what we do well and work to sustain those elements.
- We will be transparent about reporting and addressing issues that arise from the survey. We will take specific, concrete actions, report back regularly to faculty, staff, and students, and hold each other to a high, growth-minded standard.
- Department thresholds for specific actions will be established based on the information that arises from the survey results.
- Even a single instance of misconduct, abuse, or discrimination requires an appropriate and timely response.
- We anticipate issues may ebb and flow, and that some interventions may be effective while others may be well-intentioned but not prove effective. We will therefore re-evaluate our efforts and the departmental climate on a regular basis. This survey will be conducted every three years, with the expectation that our efforts will necessarily be on-going.

## Confidentiality and Anonymity

***Responses will be anonymous to encourage candid responses.***

However, we recognize that certain circumstances or demographic information may make respondents identifiable. Therefore, we clarify: questions may be skipped entirely and the results will only be presented in aggregate.

***To ensure anonymity and your candid responses, we will take the following actions:***

- Some demographic information (e.g., role/position in department) will be required, in order to pose you appropriate questions. All other demographic questions will be optional.
- Some demographics will only be reported as an aggregate of groups historically marginalized or excluded from STEM, including individuals who self-identify as nonbinary, LGBTQ+, citizens of countries other than the USA, and cultural and linguistic minorities.
- Any demographics which have fewer than 10 respondents will also be reported in an aggregate fashion.
- Analyses of raw data will be conducted by Gabe Barrile (recently graduated PhD student, Chalfoun and Walters Labs) and Justine Becker (postdoc, Merkle Lab). These individuals have signed a statement of confidentiality (see here [N.B. “here” was hyperlinked to a signed version available to survey participants]) which will be included in the survey report we prepare.

Future iterations of this survey will include similar practices, with modifications to further protect confidentiality as needed.

## Informed Consent

As a member of the UW Zoology and Physiology Department, you play a key role in the department’s operations and impacts. Thus, we are asking you to participate in this survey as we try to learn more about your experiences in the department.

We will use the results of this survey to develop priorities and an action plan for enhancing the department’s operations on many fronts. We will report back to the department and may publish a methods paper to share our process with other departments who may also want to do climate surveys. No identifying information will be included in any reports or publications.

Completing this survey indicates your consent as a participant in this study insofar as your responses will be analyzed.

*Anonymity of respondents:* Data collected is anonymous. No individual identifiers will be collected.

*Voluntary participation and optional response to individual questions:* Participating in this study is voluntary. Your refusal to participate will involve no penalty or loss of benefits to which you are otherwise entitled, and you may discontinue participation at any time. If at any time you choose to withdraw from this study, you may do so by closing out of the browser window. Also, once you’ve started the survey you do not have to answer all of the questions. While we encourage you to answer all of the questions, you may leave questions blank at any time and continue with the survey.

*More information about the survey:* This survey has been approved by the University of Wyoming Institutional Review Board. If you have any questions or concerns regarding this research study, feel free to contact Bethann Garramon Merkle (bmerkle@uwyo.edu) or Michael Dillon (michael.dillon@uwyo.edu).

By proceeding with this survey, you give your informed consent and verify that you are 18 years of age or older. In the actual survey, we ask you to confirm both of these before commencing the survey.

## Definitions & Acronyms

The following terms and acronyms are used in the survey. We provide definitions in the survey and provide them here for your reference.

**Ableist/non-ableist:** discrimination or prejudice against individuals with disabilities (from [Merriam-Webster online dictionary](https://www.merriam-webster.com/dictionary/ableism))

**Ageist/non-ageist:** prejudice or discrimination against a particular age-group and especially the elderly (from [Merriam-Webster online dictionary](https://www.merriam-webster.com/dictionary/ageism))

**Climate:** The overall nature of one’s experience in a setting/the vibe or nature of the setting as you experience it. For the survey, we are interested in your experiences in the ZP Department.

**Discrimination:** “For many people, discrimination is an everyday reality. Discrimination is the unfair or prejudicial treatment of people and groups based on characteristics such as race, gender, age or sexual orientation.” Definition from the [American Psychological Association](https://www.apa.org/topics/racism-bias-discrimination/types-stress).

**Implicit/explicit expectations:** These definitions are from [the Merriam-Webster online dictionary](https://www.merriam-webster.com/).

- ***Implicit:*** 1a: capable of being understood from something else though unexpressed. Implied. An implicit assumption. 1b: present but not consciously held or recognized (implicit attitudes, implicit racism).
- ***Explicit:*** 1a: fully revealed or expressed without vagueness, implication, or ambiguity: leaving no question as to meaning or intent. Explicit instructions
- ***Examples:*** To clarify, implicit expectations might be "my supervisor works over holidays," while explicit expectations might be "my supervisor has a written policy defining the length of the work day."

**JEDI:** Justice, equity, diversity, and inclusion; this acronym is rising in use (compared to DEI, diversity, equity, and inclusion) due to the growing recognition that justice is an essential component of DEI work.

**Junior/senior:** We use junior to refer to employees who are new-ish in their role (1-3 years for staff, 1-6 years for faculty of all types). Senior employees are those who have been with the department longer than these thresholds.

**Mental health and wellbeing:** These definitions are derived from [mentalhealth.gov](https://www.mentalhealth.gov/basics/what-is-mental-health) and [the CDC](https://www.cdc.gov/hrqol/wellbeing.htm#three).

- ***Mental health:*** Mental health includes our emotional, psychological, and social well-being. It affects how we think, feel, and act. It also helps determine how we handle stress, relate to others, and make choices. Mental health is important at every stage of life, from childhood and adolescence through adulthood.
- ***Wellbeing:*** There is no consensus around a single definition of well-being, but there is general agreement that at minimum, well-being includes the presence of positive emotions and moods (e.g., contentment, happiness), the absence of negative emotions (e.g., depression, anxiety), satisfaction with life, fulfillment and positive functioning. In simple terms, well-being can be described as judging life positively and feeling good. For public health purposes, physical well-being (e.g., feeling very healthy and full of energy) is also viewed as critical to overall well-being.

**Qualtrics:** A survey platform well-regarded in research settings for effective, user-friendly survey delivery and the capacity for respondents to be totally anonymous. We use Qualtrics through a license that the University of Wyoming offers all UW affiliates.

**R&P:** Review and promotion, for staff roles

**T&P/RT&P:** Tenure and promotion, for faculty on the tenure track. Retention, tenure, and promotion, for non-tenure-track faculty.

**Sense of Belonging:** Belonging is the feeling of security and support when there is a sense of acceptance, inclusion, and identity for a member of a certain group. It is when an individual can bring their authentic self to work. When employees feel like they don’t belong at work, their performance and their personal lives suffer. Creating genuine feelings of belonging for all is a critical factor in improving engagement and performance, along with retention. (Adapted from Cornell University: <https://diversity.cornell.edu/belonging/sense-belonging>)

**Service, departmental service:** Service is a formal expectation in some department roles’ job descriptions. ZP recognizes three categories of service: (1) program and university service; (2) contributions to professional activities; (3) contributions to community service. For more information, see [this expectations document](https://drive.google.com/file/d/1cgnx3rQnE50Gx9iEAmuJ6uQM1on1GjBn/view?usp=sharing) or [this peer-reviewed paper](https://www.dropbox.com/s/812kcpbcifjvb9r/Macfarlane%202007%20-%20Defining%20and%20Rewarding%20Academic%20Citizenship%20The%20implications%20for%20university%20promotions%20policy.pdf?dl=0) on academic citizenship (aka service).

**UW:** University of Wyoming

**Underrepresented Minorities (URM):** Sometimes also written as Underrepresented and Diverse Minorities (URDM), this term refers to individuals or demographics who are less present in an environment than their presence in a given population. As the National Science Foundation (NSF) puts it, “The representation of certain groups of people in science and engineering (S&E) education and employment differs from their representation in the U.S. population. Women, persons with disabilities, and three racial and ethnic groups—blacks, Hispanics, and American Indians or Alaska Natives—are underrepresented in S&E.” This definition informs our use of URM throughout the climate survey. For more information, see [this explainer](https://www.nsf.gov/statistics/2017/nsf17310/digest/introduction/) from NSF.

**ZP or Z&P:** Zoology and Physiology Department
