# Supplementary material for: Equity, community, and accountability: Leveraging a department-level climate survey as a tool for action
Source: PLoS One. 2023 Aug 17;18(8):e0290065. doi: 10.1371/journal.pone.0290065 (PMC10434968; doi:10.1371/journal.pone.0290065)
Supplement: S4 File — (DOCX) [file pone.0290065.s004.docx]

**Supplement 4**

**Journal Name:** *PLOS ONE*

**Manuscript Title**: Equity, community, and accountability: leveraging a department-level climate survey as a tool for action

**List of Authors:**

Gabriel M. Barrile, Riley F. Bernard, Rebecca C. Wilcox, Justine A. Becker, Michael E. Dillon, Rebecca R. Thomas-Kuzilik, Sara P. Bombaci, and Bethann Garramon Merkle

**Corresponding Author Email:** [gbarrile15@gmail.com](mailto:gbarrile15@gmail.com)

**Supplement 4 – Complete results from the exploratory factor analysis**

Most categories on our climate survey consisted of several individual questions (see Supplement 1 for the full survey). Given the similarity of questions within the same category, we expected that fewer underlying constructs may better explain the observed responses. Therefore, to reduce the complexity of responses, we conducted factor analyses to aggregate multiple questions into one or more indices. We ultimately conducted factor analyses for questions in three of the five workplace climate categories, including importance of DEIJ, sense of belonging, and work-life balance.

To perform factor analysis, we first assessed correlation matrices for each topic to determine the number of factors to extract. Using these factor numbers, we conducted minimum residual factor analyses using the *fa*() function within the ‘psych’ package (Revelle, 2022) in Program R version 4.1.1 (R Core Team 2021). Factors were rotated to an oblique solution using the *oblimin*() function from the ‘GPArotation’ package (Bernaards and Jennrich, 2005). To ensure that we used the appropriate number of dimensions to represent the correlation matrix, we continued extracting factors until the chi-square of the residual matrix was not significant. We next obtained factor scores from each analysis using the factor.*scores*() function within the ‘psych’ package (Grice, 2001). We then evaluated the standardized loadings of factor scores (based on the correlation matrix) to determine which individual questions from each topic belonged within the same index. Finally, we averaged responses within each index to obtain mean scores for each aggregate grouping of individual questions. Below we report the complete results of our exploratory factor analyses, including factor loadings on each factor score.

*Importance of DEIJ*

To explore the factorial structure of questions within the *Importance of DEIJ* section, we conducted a factor analysis on the third set of questions in that section. We chose this analytical structure because the third set of questions in this section included eight questions that were similar in nature. All eight questions were subjected to an exploratory factor analysis with oblique rotation (oblimin). The Kaiser-Meyer-Olkin measure (using the *KMO*() function in the ‘psych’ package) veriﬁed the sampling adequacy for the analysis: KMO = 0.80. Further, Bartlett’s test (using the *cortest.bartlett*() function in the ‘psych’ package) indicated that the correlation structure was adequate for factor analysis: ꭓ^2^_(28)_ = 520.11, p < 0.001. Factor analysis yielded a three-factor solution as the best ﬁt for the data. The results are presented in Table 1.

**Table 1.** Standardized loadings based on the correlation matrix of eight questions in the *Importance of DEIJ* category from a climate survey administered to the Department of Zoology and Physiology at the University of Wyoming during the fall of 2021. Each question contains a unique code. For instance, DEIJ.3–4 signifies the fourth question in the third set of questions in the category, whereas DEIJ.3–6 signifies the sixth question in the same third set of questions.

| Question | Factor 1 | Factor 2 | Factor 3 |  |
| --- | --- | --- | --- | --- |
| DEIJ.3–4 | **0.51** | –0.07 | 0.34 |  |
| DEIJ.3–6 | **0.84** | –0.02 | 0.10 |  |
| DEIJ.3–7 | **0.49** | 0.00 | 0.38 |  |
| DEIJ.3–8 | **1.02** | 0.01 | –0.08 |  |
| DEIJ.3–1 | 0.02 | **0.65** | –0.21 |  |
| DEIJ.3–2 | 0.08 | **0.80** | –0.06 |  |
| DEIJ.3–3 | –0.05 | **0.96** | 0.10 |  |
| DEIJ.3–5 | 0.03 | –0.01 | **0.85** |  |

*Notes:* Factors were extracted using the minimum residual solution and rotated using the oblimin method. Loadings included in each factor dimension appear in bold.

*Sense of belonging*

To explore the factorial structure of questions within the *Sense of belonging* section, we conducted two separate factor analyses: one on the first set of questions in that section and the other on the third set of questions in that section. We chose this analytical structure because both sets of questions contained nine questions of a similar nature, yet the sets themselves were quite dissimilar (i.e., questions were similar within each set, but different between sets). Each set of questions were subjected to an exploratory factor analysis with oblique rotation (oblimin). The Kaiser-Meyer-Olkin measure veriﬁed the sampling adequacy for both analyses: KMO = 0.83 for the first set and 0.85 for the third set. Further, Bartlett’s test indicated that the correlation structure was adequate for both factor analyses: ꭓ^2^_(36)_ = 347.92, p < 0.001 for the first set and ꭓ^2^_(36)_ = 558.24, p < 0.001 for the third set. Factor analysis yielded a three-factor solution as the best ﬁt for the data in both sets of questions. The results for the first set are presented in Table 2 and the results for the second set are presented in Table 3.

**Table 2.** Standardized loadings based on the correlation matrix for the first set of questions (*n* = 9) in the *Sense of Belonging* category from a climate survey administered to the Department of Zoology and Physiology at the University of Wyoming during the fall of 2021. Each question contains a unique code. For instance, SB.1–1 in the table signifies the first question in the first set of questions within the *Sense of Belonging* category, whereas SB.1–2 signifies the second question in the same first set of questions.

| Question | Factor 1 | Factor 2 | Factor 3 |  |
| --- | --- | --- | --- | --- |
| SB.1–1 | **0.56** | –0.10 | –0.01 |  |
| SB.1–2 | **0.61** | –0.19 | 0.31 |  |
| SB.1–3 | **0.76** | –0.15 | 0.00 |  |
| SB.1–4 | **0.73** | –0.10 | –0.14 |  |
| SB.1–5 | **0.86** | 0.13 | –0.08 |  |
| SB.1–6 | –0.09 | **0.33** | 0.26 |  |
| SB.1–8 | 0.01 | **0.96** | 0.06 |  |
| SB.1–9 | –0.22 | **0.38** | 0.04 |  |
| SB.1–7 | –0.04 | 0.06 | **0.96** |  |

*Notes:* Factors were extracted using the minimum residual solution and rotated using the oblimin method. Loadings included in each factor dimension appear in bold.

**Table 3.** Standardized loadings based on the correlation matrix for the third set of questions (*n* = 9) in the *Sense of Belonging* category from a climate survey administered to the Department of Zoology and Physiology at the University of Wyoming during the fall of 2021. Each question contains a unique code. For instance, SB.3–2 in the table signifies the second question in the third set of questions within the *Sense of Belonging* category, whereas SB.3–4 signifies the fourth question in the same third set of questions.

| Question | Factor 1 | Factor 2 | Factor 3 |  |
| --- | --- | --- | --- | --- |
| SB.3–2 | **0.72** | –0.10 | 0.30 |  |
| SB.3–4 | **0.68** | 0.02 | 0.21 |  |
| SB.3–5 | **0.94** | 0.05 | –0.05 |  |
| SB.3–6 | **0.83** | 0.08 | –0.11 |  |
| SB.3–7 | 0.02 | **0.75** | 0.05 |  |
| SB.3–8 | –0.13 | **0.72** | 0.19 |  |
| SB.3–9 | 0.13 | **0.85** | –0.05 |  |
| SB.3–1 | 0.25 | –0.14 | **0.41** |  |
| SB.3–3 | 0.08 | 0.16 | **0.83** |  |

*Notes:* Factors were extracted using the minimum residual solution and rotated using the oblimin method. Loadings included in each factor dimension appear in bold.

*Work-life balance*

To explore the factorial structure of questions within the *Work-life balance* section, we conducted three separate factor analyses: one on the first set of questions in that section, another on the second set of questions in that section, and the last on the third set of questions in that section. We chose this analytical structure because questions were similar within each set, but different between sets. Each set of questions were subjected to an exploratory factor analysis with oblique rotation (oblimin). The first factor analysis aggregated the only two questions in the first set of questions into one factor index (standardized loadings for both questions = 0.81). Similarly, the second factor analysis aggregated the only two questions in the second set of questions into one factor index (standardized loadings for both questions = 0.78).

The third set contained four questions. The Kaiser-Meyer-Olkin measure veriﬁed the sampling adequacy for this analysis: KMO = 0.76. Further, Bartlett’s test indicated that the correlation structure was adequate for factor analysis: ꭓ^2^_(6)_ = 457.72, p < 0.001. Factor analysis yielded a one-factor solution as the best ﬁt for the data. The results are presented in Table 4.

**Table 4.** Standardized loadings based on the correlation matrix for the third set of questions (*n* = 4) in the *Work-life balance* category from a climate survey administered to the Department of Zoology and Physiology at the University of Wyoming during the fall of 2021. Each question contains a unique code. For instance, WLB.3–1 signifies the first question in the third set of questions within the *Work-life balance* category, whereas WLB.3–2 signifies the second question within the same third set of questions.

| Question | Factor 1 |  |
| --- | --- | --- |
| WLB.3–1 | **0.83** |  |
| WLB.3–2 | **0.86** |  |
| WLB.3–3 | **0.90** |  |
| WLB.3–4 | **0.92** |  |

*Notes:* Factors were extracted using the minimum residual solution and rotated using the oblimin method. Loadings included in each factor dimension appear in bold.

**Literature Cited in Supplement 4**

Bernaards, C. A., and Jennrich, R. I. (2005). Gradient projection algorithms and software for arbitrary rotation criteria in factor analysis. *Educational and Psychological Measurement*, *65*(5), 676-696. https://doi.org/10.1177/0013164404272507

Grice, J. W. (2001). Computing and evaluating factor scores. *Psychological Methods*, *6*(4), 430. https://doi.org/10.1037/1082-989X.6.4.430

R Core Team. (2021). R: A language and environment for statistical computing. R Foundation for Statistical Computing, Vienna, Austria. www.R-project.org

Revelle, W. (2022). psych: Procedures for Personality and Psychological Research, Northwestern University, Evanston, Illinois, USA, https://CRAN.R-project.org/package=psych Version = 2.2.5.
