# Supplementary material for: Equity, community, and accountability: Leveraging a department-level climate survey as a tool for action
Source: PLoS One. 2023 Aug 17;18(8):e0290065. doi: 10.1371/journal.pone.0290065 (PMC10434968; doi:10.1371/journal.pone.0290065)
Supplement: S5 File — (DOCX) [file pone.0290065.s005.docx]

**Supplement 5**

**Journal Name:** *PLOS ONE*

**Manuscript Title**: Equity, community, and accountability: leveraging a department-level climate survey as a tool for action

**List of Authors:**

Gabriel M. Barrile, Riley F. Bernard, Rebecca C. Wilcox, Justine A. Becker, Michael E. Dillon, Rebecca R. Thomas-Kuzilik, Sara P. Bombaci, and Bethann Garramon Merkle

**Corresponding Author Email:** [gbarrile15@gmail.com](mailto:gbarrile15@gmail.com)

**Supplement 5 – Employee category-specific questions**

Here we report on questions asked specifically to individual employee categories: faculty, graduate students, and staff. We report in detail on the closed-response questions. However, to preserve anonymity of respondents, we only report aggregate results of open-response questions.

*Faculty-specific questions*

All 26 survey respondents who identified themselves as faculty completed the faculty-specific questions. Overall, faculty reported a high quality of life working in the department (e.g., very comfortable and supported) and perceived interdepartmental collaborations as a strength. However, faculty felt mixed about department resources, support, and issues regarding service (e.g., departmental, university, professional).

Overall, faculty felt comfortable providing their perspective to faculty of the same rank (92%), a higher rank (88%), or faculty who have been in the department for the same amount of time (92%) or longer (92%). Further, few felt they should defer to more senior faculty in department decisions (12%). Faculty felt like they regularly interact with faculty of the same rank (92%), higher rank (73%), or individuals in the department who are not faculty (85%). Faculty agreed that they have colleagues in the department with whom they could/have collaborate(d) (92%) and that collaboration within the department was encouraged (69%). Moreover, faculty felt comfortable recommending departmental colleagues to students (e.g., mentoring, thesis/dissertation committees, collaborations; 88%) (Figure S1).

Regarding the retention, tenure, and promotion process, faculty generally thought that expectations were clear/transparent (85% agree), fair (65% agree), on par with other institutions (62% agree), and that peer evaluation was valued by the department (62% agree). However, barely more than half considered retention, tenure, and promotion expectations to be consistently applied (54% agree) (Figure S1).

Results were mixed regarding how faculty felt about department resources and support. While many thought that mentoring committees were a useful resource for pre-tenured faculty (62%), few believed that the mentoring committees had specific goals, objectives, and outcomes (27%), suggesting that this process would be improved by having more structure. Whereas faculty thought that the department provided support for teaching (54%), fewer agreed that there was support for onboarding and mentoring of students (44%), or resources for grant writing (15%) (Figure S1). Regarding service, while faculty agreed that expectations of pre-tenure faculty were reasonable (62%), half thought that a few faculty did most of the service within the department (50%), and few believed that department service was equitable among faculty within the department (12%).

More encouraging open-response comments from faculty addressed topics including the potential to have a more cohesive department, multidisciplinarity as a departmental strength, and valuing the climate of the department over a more prestigious institution. Less encouraging open-response comments addressed topics including how to improve faculty mentoring committees, service inequities among faculty, frustration over lip-service encouraging collaboration without substantial support, reminders to consider that seniority is not the only power dynamic at play, and calls for more functional retention, tenure, and promotion processes considering the multidisciplinarity of the department.

*Graduate student questions*

All 67 survey respondents who identified themselves as graduate students completed the graduate student-specific questions. Graduate students agreed that their advisors encouraged them to attend professional conferences (90%), present research (87%), publish as a co-author (74%) and first author (88%), and develop strong relationships with academic/professional mentors (60%). By contrast, graduate students felt that their advisors were less likely to encourage them to serve on committees (19%), participate in outreach (46%), participate in DEIJ activities (24%), or mentor undergraduate students (44%) (Figure S2).

Most graduate students thought that their advisors performed their mentoring/advising roles well (82%); however, there is room for improvement and even reports of concerning supervisor behavior (discussed in detail in the Discussion of the main text). Most graduate students felt that their advisors were knowledgeable about the university and department degree requirements (63%), helped secure financial support for graduate work (81%), served as an advocate when necessary (79%), provided constructive feedback on research (81%), returned work promptly (74%), provided guidance through major academic milestones (74%), and created a supportive and productive lab culture (69%). However, while most graduate students felt that advisors promoted their professional development (79%), they were less confident in their advisors’ ability to provide helpful advice about career paths within (63% agreed) or outside academia (51% agreed) or generally assist them in searching for post-graduation employment (40% agreed).

Most graduate students felt like they received accurate information about funding, stipend, and benefits when hired (72%), that their advisors provided additional funding when needed (e.g., summer salary, conference travel; 75%), and that advisors helped them apply for funding via grants or scholarships (68%). Many were issued a personal university purchasing card (72%) and few encountered situations where their advisors expected them to pay for out-of-pocket costs related to research and be reimbursed later (12%). However, few believed that their graduate stipend was sufficient to cover their living expenses (e.g., rent, food, medical expenses; 26%), and very few felt that their compensation was fair for the work that they did (19%). Furthermore, almost half (43%) of students had extra financial obligations (e.g., supporting family members/dependents, paying off debts), suggesting that they could have been experiencing further financial stress. Lastly, many believed that their compensation is on par with comparable programs elsewhere (43%), suggesting that low pay was perceived to be a common issue for graduate students across institutions.

More encouraging open-response comments from graduate students addressed topics including positive overall experiences, supportive peers and/or advisors, lack of competitiveness in department culture, and appreciation that faculty were advocating for higher wages. Less encouraging open-response comments addressed topics including poverty-level wages, not being allowed to take outside jobs, dumpster diving as a common activity among students, toxic supervisory relationships, desire for clearer degree requirements, need for comprehensive health care benefits, and lack of support/knowledge among faculty for careers outside of academia.

*Staff-specific questions*

Of the 16 survey respondents who identified themselves as staff, nine answered the staff-specific questions. Staff generally felt valued by others within the department. Specifically, staff felt valued by colleagues (69%), supervisors (69%), the department (56%), graduate students (50%), faculty (44%), and undergraduate students (38%). Further, few staff felt concerned about taking time off (38%). In contrast, few staff believed that they were fairly compensated for their work (30%), empowered to do what they do best (44%), or felt a sense of belonging in the department (27%). None of the staff respondents felt that they had a voice in the decisions that affect the future of the department (0%). Further, few felt like they had any room for professional development within the department (44%), or the university (38%) (Figure S3).

More encouraging open-response comments from staff indicated that department leadership was perceived positively. Less encouraging open-response comments addressed topics including frustration with leadership, lack of mentoring, advancement, and professional development opportunities for staff, desire for work-from-home flexibility, a need for relocation support for new hires, desire for re-allocation of funding to support faculty and staff (not just buildings and upper administration), and better pay for staff.

**Figures**


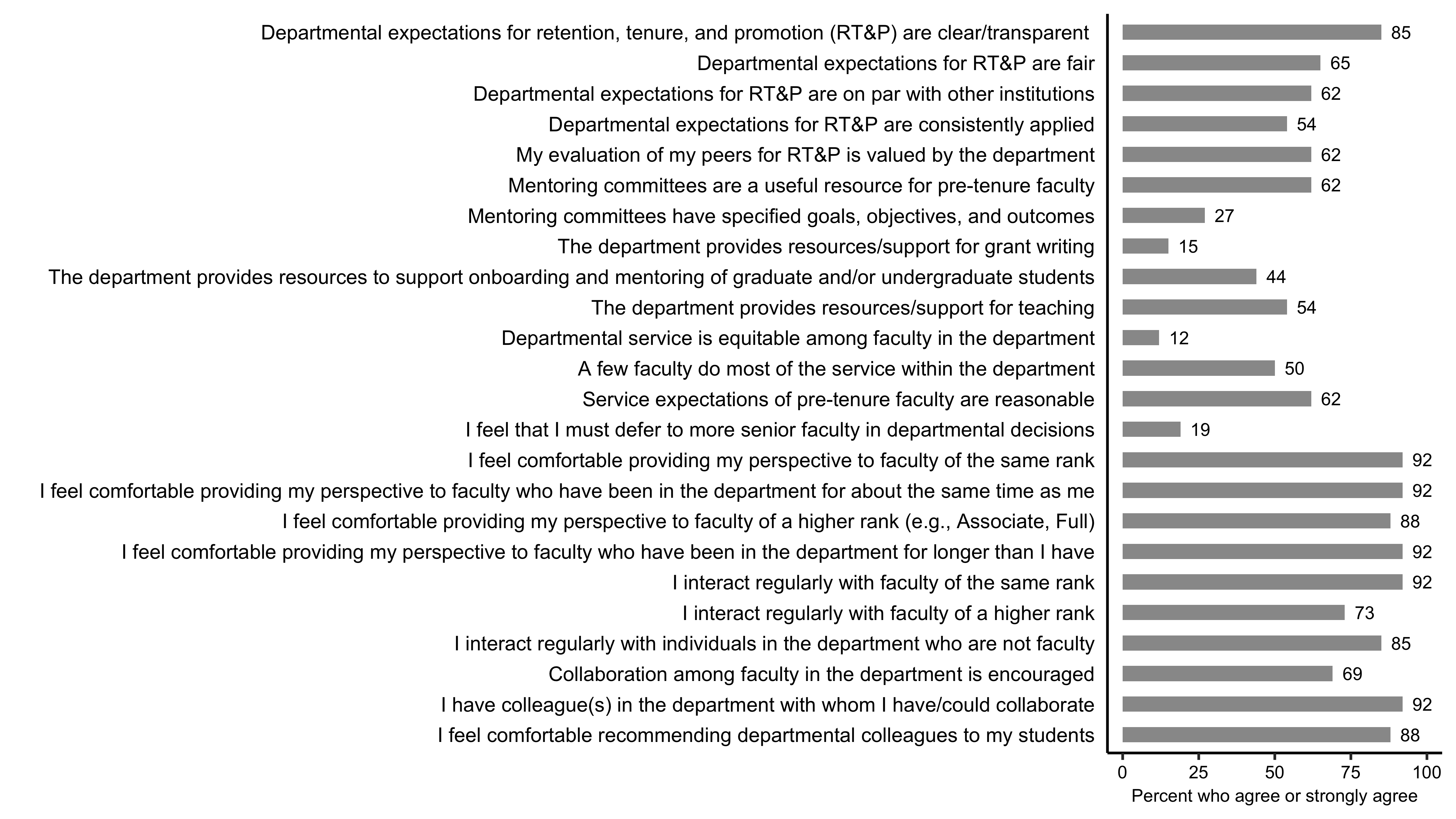


**Figure S1**. Percent of faculty who agreed or strongly agreed with faculty-specific questions.


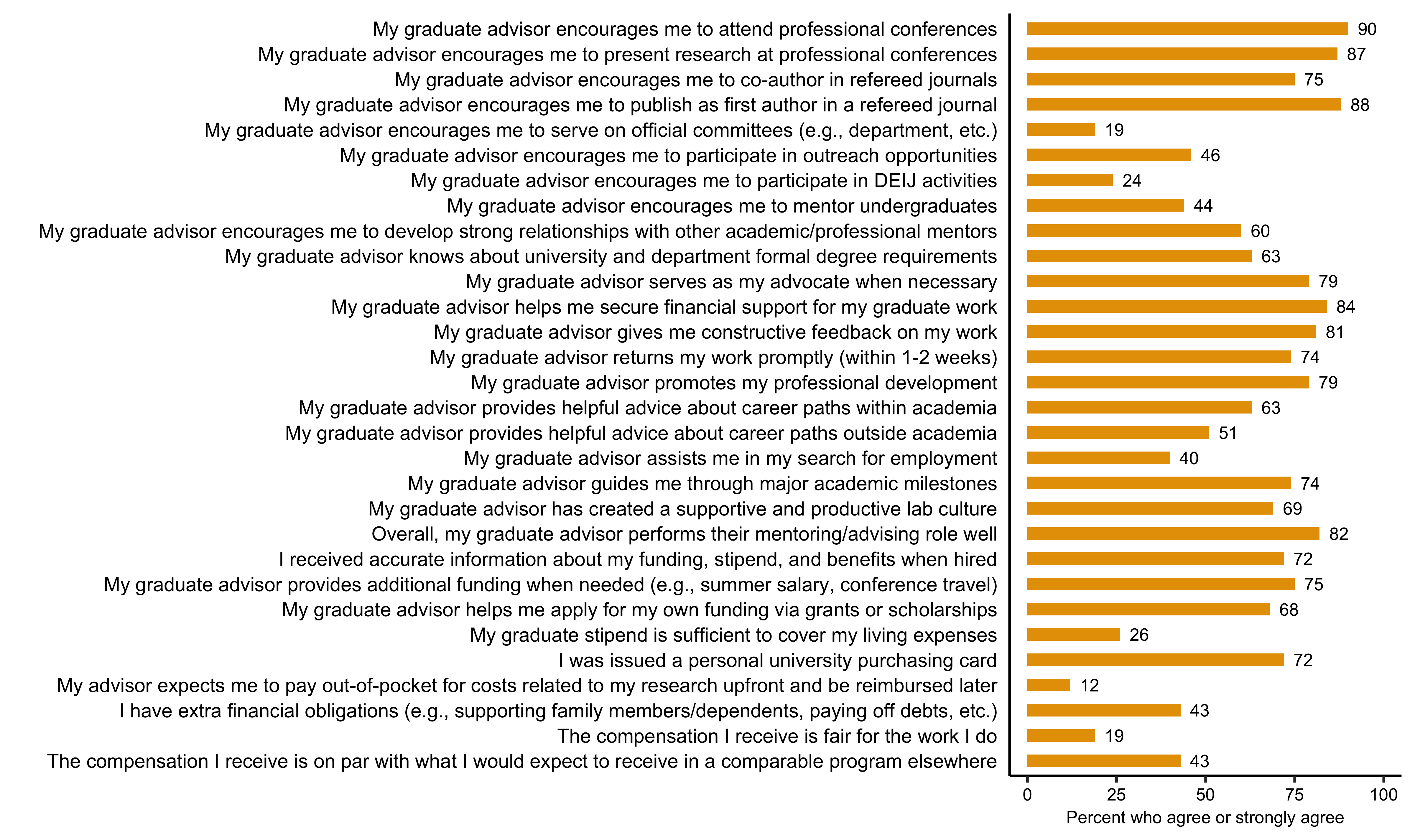


**Figure S2.** Percent of graduate-students who agreed or strongly agreed with graduate student-specific questions.


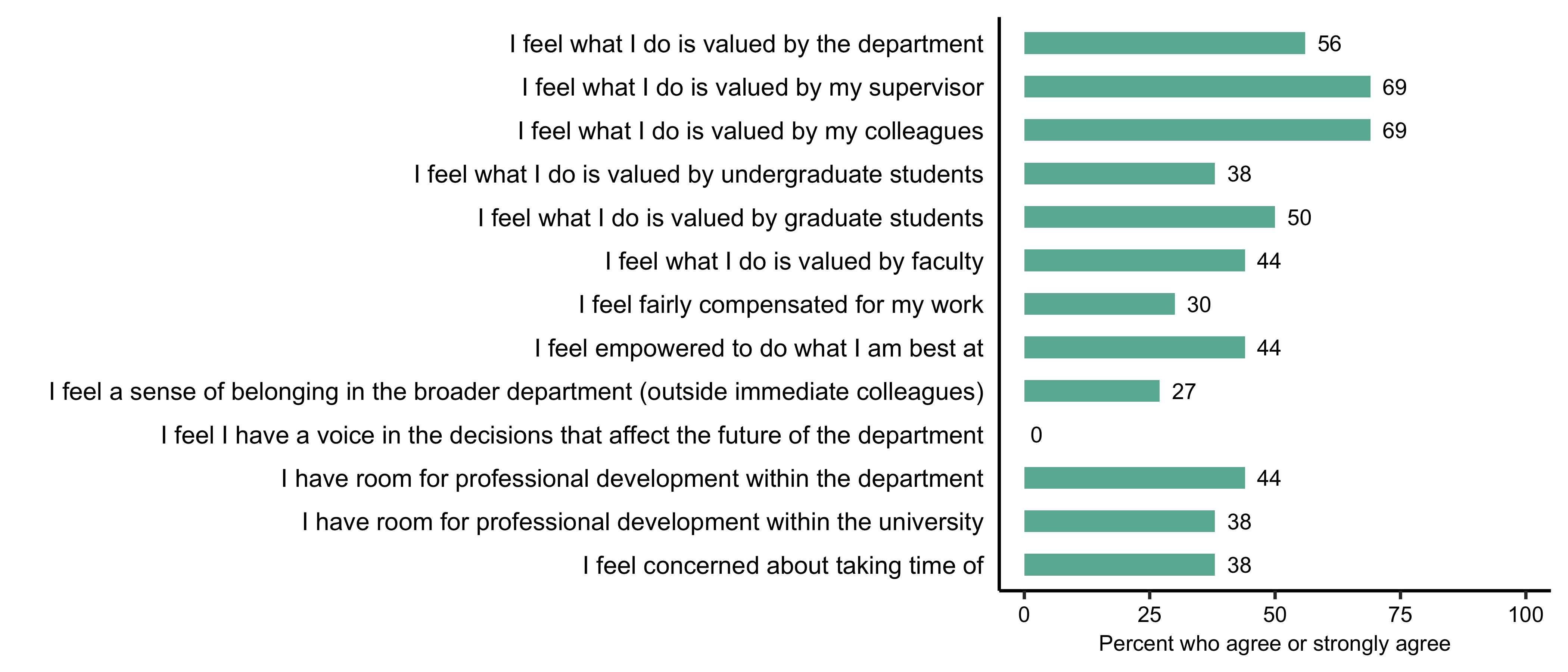


**Figure S3.** Percent of staff who agreed or strongly agreed with staff-specific questions.
